# Advances in Molecular Imaging of VEGFRs: Innovations in Imaging and Therapeutics

**DOI:** 10.3390/ijms26115373

**Published:** 2025-06-04

**Authors:** Hanieh Karimi, Sarah Lee, Wenqi Xu, Sigrid A. Langhans, David K. Johnson, Erik Stauff, Heidi H. Kecskemethy, Lauren W. Averill, Xuyi Yue

**Affiliations:** 1 Department of Radiology, Diagnostic and Research PET/MR Center, Nemours Children’s Health, Wilmington, DE 19803, USA; hanieh.karimi@nemours.org (H.K.); slee43@wm.edu (S.L.); wenqi600991@outlook.com (W.X.); erik.stauff@nemours.org (E.S.); heidi.kecskemethy@nemours.org (H.H.K.); lauren.averill@nemours.org (L.W.A.); 2 Diagnostic & Research PET/MR Center, Nemours Children’s Health, Delaware, Wilmington, DE 19803, USA; sigrid.langhans@nemours.org; 3 Division of Neurology, Nemours Children’s Health, Delaware, Wilmington, DE 19803, USA; 4 Computational Chemical Biology Core, University of Kansas, Lawrence, KS 66047, USA; dkjohnson@ku.edu; 5 Department of Radiology, Thomas Jefferson University, Philadelphia, PA 19107, USA; 6 Department of Psychological and Brain Sciences, University of Delaware, Newark, DE 19716, USA

**Keywords:** VEGFRs, nuclear medicine imaging, radiopharmaceuticals, angiogenesis, lymphangiogenesis, targeted therapy

## Abstract

Vascular endothelial growth factor receptors (VEGFRs) are key regulators of angiogenesis, lymphangiogenesis, and vascular permeability, playing essential roles in both physiological and pathological processes. The VEGFR family, including VEGFR-1, VEGFR-2, and VEGFR-3, interacts with structurally related VEGF ligands (VEGFA, VEGFB, VEGFC, VEGFD, and placental growth factor [PlGF]), activating downstream signaling pathways that mediate critical cellular processes, including proliferation, migration, and survival. Dysregulation of VEGFR signaling has been implicated in numerous diseases, such as cancer, cardiovascular conditions, and inflammatory disorders. Targeting VEGFRs with radiopharmaceuticals, such as radiolabeled peptides, antibodies, and specific tracers like ^64^Cu-bevacizumab and ^89^Zr-ramucirumab, has emerged as a powerful strategy for non-invasive imaging of VEGFR expression and distribution in vivo. Through positron emission tomography (PET) and single-photon emission computed tomography (SPECT), these targeted tracers enable real-time visualization of angiogenic and lymphangiogenic activity, providing insights into disease progression and therapeutic responses. This review explores the current advances in VEGFR-targeted imaging, focusing on the development of novel tracers, radiolabeling techniques, and their in vivo imaging characteristics. We discuss the preclinical and clinical applications of VEGFR imaging, highlight existing challenges, and provide perspectives on future innovations that could further enhance precision diagnostics and therapeutic monitoring in angiogenesis and lymphangiogenesis-driven diseases.

## 1. Introduction

**Figure 1:**
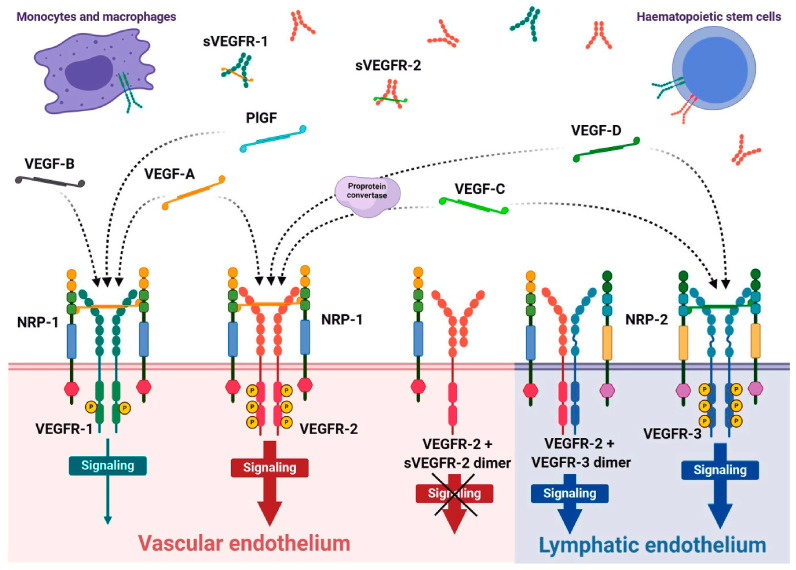
VEGF receptors and signaling pathways. Adopted from Masłowska, K. et al., Cancers, 2021, DOI: 10.3390/cancers13051072. Licensed under CC BY 4.0 [6]. NRP 1, co-receptor neuropilin 1; NRP 2, co-receptor neuropilin 2; VEGFR refers to transmembrane receptor tyrosine kinases, while sVEGFR refers to the soluble form of the VEGFR family.

Vascular endothelial growth factor receptors (VEGFRs) are membrane-bound receptor tyrosine kinase proteins critical for the regulation of angiogenesis, vascular permeability, lymphangiogenesis, the differentiation and proliferation of cells, and the formation of tissues [1]. The VEGFR family consists of three main receptors (VEGFR-1, VEGFR-2, VEGFR-3) that bind to five corresponding structurally related VEGF ligands (VEGFA, VEGFB, VEGFC, VEGFD, and placental growth factor [PlGF]). Each ligand–receptor interaction activates distinct signaling pathways that promote cellular processes such as proliferation, migration, and survival, which are essential for vascular and lymphatic system development and maintenance [2,3]. VEGFR-2, for instance, is primarily responsible for mediating angiogenic responses, while VEGFR-3 is more involved in lymphangiogenesis (Figure 1) [4,5,6]. The precise regulation of these signaling interactions is crucial for normal physiological functions; however, dysregulation of the signal pathways is linked to a variety of diseases, such as cancer, inflammatory disorders, and cardiovascular diseases [7]. Due to their pivotal role in vascular and lymphatic biology, VEGFRs and their ligands are major targets for diagnostic and therapeutic interventions aimed at treating these conditions [8].

Radiopharmaceuticals targeting VEGFRs offer a powerful approach to visualize VEGFR distribution and expression levels in vivo, providing critical insights into the angiogenic activity of tumors and other pathological tissues [9,10,11]. For example, bevacizumab and ramucirumab are two Food and Drug Administration (FDA)-approved antibody drugs for cancer treatment. Their radiolabeled derivatives, ^64^Cu-bevacizumab, which binds VEGF-A, or ^89^Zr-ramucirumab, which specifically targets VEGFR-2, have been used for cancer detection by targeting the VEGF family [12,13,14]. By using radioisotopes attached to VEGFR-specific ligands, these radiopharmaceuticals enable non-invasive imaging through various modalities such as positron emission tomography (PET) and single-photon emission computed tomography (SPECT) [15]. Once administered, these targeted radiopharmaceuticals bind selectively to VEGFRs expressed on lymphatic vessels or vascular endothelial cells within tissues, allowing for real-time mapping of receptor density and localization [6,16]. This strategy not only helps to assess the extent of VEGFR involvement in angiogenesis but also aids in evaluating the response to anti-angiogenic therapies by tracking changes in VEGFR expression over time [16]. High-sensitivity VEGFR imaging can thereby assist in patient stratification, monitoring disease progression, and tailoring treatment strategies. Therefore, VEGFR-targeted radiopharmaceuticals provide a valuable tool in precision oncology and the management of angiogenesis-driven diseases [17].

This review focuses on imaging the VEGF family in different disease conditions, encompassing both bench research and clinical investigations. We specifically highlight the recent development of various VEGFR-targeted tracers, examining aspects like radiolabeling methods, binding affinity, selectivity, and in vivo imaging characteristics. Finally, we address the current limitations of VEGFR imaging and discuss future applications and advancements in this field.

### 1.1. VEGFR Family Role in Normal and Diseased Conditions

The VEGFR family is crucial in regulating angiogenesis, lymphangiogenesis, and vascular permeability. Angiogenesis is a process in the formation of new blood vessels from pre-existing ones, while lymphangiogenesis refers to the formation of new lymphatic vessels. The family includes three main receptors: VEGFR-1 (also known as Flt-1), VEGFR-2 (KDR/Flk-1), and VEGFR-3 (Flt-4) [18,19]. Each of these receptors specifically interacts with one or multiple ligands, including VEGF-A, VEGF-B, VEGF-C, and VEGF-D, to initiate critical cell signaling pathways. These interactions are not only integral to the development and maintenance of the vascular system [20,21]; they are also essential for various physiological processes, such as endothelial cell proliferation, migration, and survival [22,23]. VEGFR-3 specifically mediates the formation of lymphatic vessels, contributing to the proper drainage of interstitial fluid and immune responses [24,25]. In the context of diseases, including many tumors, VEGFR is overexpressed, leading to enhanced angiogenesis that supports tumor growth and metastasis. Tumors secrete high levels of VEGF, which stimulates nearby endothelial cells to proliferate and form new blood vessels [8,26]. Anti-VEGF therapies (e.g., bevacizumab) aim to inhibit this process to starve tumors of their blood supply [27,28]. Abnormal VEGFR signaling can lead to atherosclerosis, hypertension, and other cardiovascular disorders due to improper angiogenesis or vascular remodeling [29]. Conditions like age-related macular degeneration and diabetic retinopathy are characterized by pathological angiogenesis driven by VEGFR signaling, leading to vision loss [30,31]. VEGFR signaling can contribute to the recruitment of inflammatory cells and enhance vascular permeability, exacerbating autoimmune diseases and chronic inflammatory conditions [32]. Dysregulation of VEGFR signaling is implicated in conditions such as diabetic nephropathy, where abnormal angiogenesis can affect kidney function [33].

VEGFR-1 is a receptor tyrosine kinase primarily expressed on endothelial cells, monocytes, and macrophages. It has a long extracellular domain with seven immunoglobulin-like (Ig-like) motifs, which are crucial for ligand binding. VEGFR-1 binds to several ligands, including VEGF-A, VEGF-B, and PlGF [34]. It has a higher affinity for VEGF-A when compared with VEGFR-2. VEGFR-1 primarily acts as a negative regulator of angiogenesis [8]. It competes with VEGFR-2 for VEGF-A binding, thereby modulating VEGF signaling. It is involved in the recruitment and differentiation of monocytes and macrophages during tissue remodeling and inflammation. Dysregulation of VEGFR-1 is associated with various conditions, including cancer, where it may promote tumor growth and metastasis by supporting angiogenesis through indirect mechanisms [35].

VEGFR-2 is the most studied receptor in the VEGFR family and is predominantly expressed on endothelial cells. It contains a shorter extracellular domain than VEGFR-1 and is crucial for mediating most VEGF-induced cellular responses [3,36]. VEGFR-2 primarily binds to VEGF-A but can also interact with VEGF-C and VEGF-D. This receptor is responsible for transducing signals that lead to endothelial cell proliferation, migration, and survival. VEGFR-2 activation promotes angiogenesis, increases vascular permeability, and enhances endothelial cell survival [8,37]. It initiates a cascade of intracellular signaling pathways, including the phosphatidylinositol 3-kinase (PI3K)/Akt and mitogen-activated protein kinase (MAPK) pathways. Overexpression or dysregulation of VEGFR-2 is commonly associated with various cancers, contributing to tumor angiogenesis and metastasis. Targeting VEGFR-2 with monoclonal antibodies and small-molecule inhibitors has become a therapeutic strategy in cancer treatment [38,39].

VEGFR-3 is mainly expressed in lymphatic endothelial cells and plays a critical role in lymphangiogenesis. It primarily binds to VEGF-C and VEGF-D, which are involved in the development and maintenance of lymphatic vessels [40,41]. Unlike VEGFR-2, it does not significantly bind to VEGF-A. Activation of VEGFR-3 promotes lymphatic endothelial cell proliferation, migration, and survival. It also supports immune responses and fluid homeostasis by regulating lymphatic drainage [5]. VEGFR-3 and its ligand VEGF-C are pivotal for the development and function of the meningeal lymphatic system, ensuring structural integrity and fluid clearance during embryonic and postnatal development [42,43,44]. These vessels facilitate cerebrospinal fluid (CSF) clearance, removing metabolic waste and proteins, including amyloid-beta, which is implicated in Alzheimer’s disease [45,46]. Impaired VEGFR-3 signaling can disrupt lymphatic drainage in the central nervous system, exacerbating neuroinflammation and disease progression. Additionally, VEGFR-3-mediated pathways support immune cell trafficking and regulate inflammation by modulating antigen-presenting cell movement to lymph nodes. These dual roles in waste clearance and immune surveillance highlight VEGFR-3 as a promising therapeutic target. Strategies to enhance VEGFR-3/VEGF-C signaling could improve outcomes in neurodegenerative diseases, multiple sclerosis, and brain injuries, with ongoing research exploring molecular mechanisms, therapeutic interventions, and advanced imaging for real-time monitoring [41,47]. Dysfunction of VEGFR-3 expression levels also contributes to tumor lymphangiogenesis and metastasis to lymph nodes. Targeting VEGFR-3 is being explored as a potential therapeutic strategy for limiting lymphatic metastasis in cancers [26,48]. Table 1 summarizes the roles of the VEGF-VEGFR family in disease conditions.

VEGFR-targeting therapies can be effectively combined with other treatment modalities, such as chemotherapy and immunotherapy. This can enhance the overall efficacy of cancer treatment regimens by addressing multiple pathways involved in tumor progression and immune evasion [22,48]. By focusing on the endothelial cells of blood vessels, these therapies can minimize damage to surrounding healthy tissues, potentially reducing side effects compared to conventional therapies [33]. Tumors often develop resistance to therapies over time. Targeting the VEGFR pathway can overcome some of these resistance mechanisms, as tumor blood supply is critical for their survival and proliferation [45]. By disrupting this supply, it can enhance the effectiveness of other treatments and delay resistance. New developments in targeting VEGFR include small molecule inhibitors, monoclonal antibodies, and RNA-based therapies, providing a range of options for therapeutic intervention [65,66].

### 1.2. Molecular Targeting of the VEGFR System Using Imaging Probes

Molecular imaging probes used for targeting the VEGF-VEGFR system are highly specialized agents that enable the visualization of angiogenesis processes critical in cancer and other diseases characterized by abnormal blood and lymphatic vessel growth. These probes, including radiolabeled antibodies, peptides, and small molecular inhibitors, are designed to bind specifically to VEGF ligands or their receptors (VEGFRs), predominantly VEGFR-1 and VEGFR-2. Once bound, they facilitate imaging through modalities like PET, SPECT, magnetic resonance imaging (MRI), and optical fluorescence, allowing non-invasive monitoring of VEGF-VEGFR expression and interactions [67]. By highlighting regions of active angiogenesis, these probes provide insights into tumor biology, help assess the efficacy of anti-angiogenic therapies and allow for the early detection of disease progression. Additionally, they are valuable in preclinical and clinical research for mapping out disturbed expression levels and distribution of the VEGF family, aiding in the development of new therapeutic strategies [68].

Radiolabeled VEGF probes targeting the various isoforms, including VEGF-A, VEGF-B, VEGF-C, VEGF-D, VEGFR-1, VEGFR-2, and VEGFR-3, are essential for advancing our understanding of angiogenesis and lymphangiogenesis in both normal physiology and pathological conditions such as cancer [6]. Each VEGFR ligand plays a distinct role in vascular and lymphatic development: VEGF-A primarily mediates blood vessel formation, while VEGF-B is involved in endothelial cell survival and stabilization processes. VEGF-C and VEGF-D are crucial for lymphangiogenesis and are associated with lymphatic endothelial cells [2,3,4,5]. By conjugating radionuclides, such as ^99m^Tc, ^18^F, ^111^In, or ^89^Zr, to antibodies or small molecules that specifically bind to VEGFR-1, VEGFR-2, or VEGFR-3, researchers can develop imaging agents capable of visualizing the spatial distribution and density of these receptors in vivo [69,70]. With imaging techniques such as PET and SPECT, these radiolabeled probes enable the non-invasive tracking of receptor expression levels, distribution, and functionality, providing valuable information about lesion microenvironments, the dynamics of vascular and lymphatic networks, and disease progression and metastasis, ultimately contributing to developing more personalized anti-angiogenic therapies targeting the VEGFR family treatment [6,68].

#### 1.2.1. Radiolabeled Small Molecules

Molecular targeting of the VEGF-VEGFR system using radiolabeled small molecules has become a pivotal strategy in diagnosing and treating cancer. Advances in radiolabeling technology have led to the development of small molecules that specifically target VEGFR receptors, offering high-resolution imaging and enhanced therapeutic efficacy [6]. Small molecule inhibitors have been used in therapeutic and imaging applications due to their pharmacokinetic properties [71]. Their low molecular weight allows for rapid tissue penetration and clearance, which results in a high tumor-to-background ratio during imaging studies [72]. Radiolabels such as positron emitters (^18^F, ^68^Ga) and gamma emitters (e.g., ^99m^Tc) are frequently used to enhance the imaging capabilities of VEGFR-targeted tracers. These molecules are engineered to have a high affinity for VEGFRs, allowing them to visualize tumors with high specificity [73].

Another advantage in developing small molecular imaging agents is their ability to cross the blood–brain barrier (BBB) and target specific neurological processes. A widely accepted guideline for designing effective small molecules is Lipinski’s Rule of Five, which predicts drug-likeness and BBB permeability. The five key parameters include a molecular weight (MW) ≤ 500 Daltons (Da), a partition coefficient (LogP) ≤ 5, no more than 5 hydrogen bond donors (OH and NH groups), no more than 10 hydrogen bond acceptors (N and O atoms), and a molar refractivity between 40 and 130. Molecules with a molecular weight under 500 Da exhibit better membrane permeability, and an optimal LogP range of 1.5–2.5 further enhances the possibility for BBB penetration [74]. Recent studies emphasize the importance of these properties in the context of brain imaging, noting that molecular size, shape, and lipophilicity significantly influence BBB transport mechanisms. Advances in ligand design and transport system targeting are improving the efficacy of small molecules, making them indispensable tools for diagnosing and studying neurological disorders [75,76].

Furthermore, small molecules can be readily radiolabeled and used as imaging agents without significant alteration of their binding properties. One example is the use of radiolabeled tyrosine kinase inhibitors (TKIs) to target VEGFRs, a key player in angiogenesis. Poot et al. [77] evaluated the versatility of TKIs as imaging agents, noting their ability to selectively bind VEGFRs while maintaining functionality upon radiolabeling with isotopes such as fluorine-18 or carbon-11 for PET imaging. These radiolabeled TKIs provide high sensitivity and specificity in detecting VEGFR expressions, offering critical insights into the angiogenic processes within tumors. The study also emphasized the clinical relevance of this approach, as VEGFR-targeted imaging facilitates not only the diagnosis of angiogenesis-driven malignancies but also the evaluation of anti-angiogenic therapies by tracking changes in receptor expression over time. This application shows the potential of radiolabeled small molecules, particularly TKIs, to advance personalized medicine by enabling precise, non-invasive monitoring of tumor vasculature and response to treatment.

Li et al. [73] developed two vandetanib analogs, a monomeric (ZD-G1) and a dimeric (ZD-G2). Both were conjugated with 1,4,7,10-tetraazacyclododecane-1,4,7,10-tetraacetic acid (DOTA) for ^64^Cu labeling to create ^64^Cu-DOTA-ZD-G1 and ^64^Cu-DOTA-ZD-G2, respectively. VEGFR-binding assays using U-87 MG cells (known to overexpress VEGFR) revealed a mean K_d_ value of 44.7 nM for the monomeric form and 0.45 nM for the dimeric form, indicating a nearly 100-fold improvement in U-87 MG tumor–bearing mice, which showed the dimeric probe was over five times greater than that of the monomeric probe across all time points, with statistical significance (*p* < 0.0001). Although ^64^Cu-DOTA-ZD-G2 showed favorable biodistribution results, further refinement could reduce liver and kidney uptake, enhancing signal specificity and minimizing radiation toxicity. These findings underscore the superior targeting capability of the dimeric probe, likely due to enhanced receptor affinity from multivalency. The dimeric structure thus offers a promising approach for effective imaging of VEGFR expression in tumors, with potential applications in both diagnostics and therapy monitoring in VEGFR-targeted treatments. Given that the probe is based on the FDA-approved drug vandetanib, it has strong potential for rapid translation into clinical settings. Further studies may validate its effectiveness in human trials, particularly for cancers with high VEGFR expression, such as glioblastomas and colorectal cancer.

Wang et al. [78] synthesized ^18^F-SU11248, a new potential PET tracer for imaging cancer tyrosine kinase activity, including VEGF, using the combined solid phase extraction (SPE)-HPLC technique. The radiolabeling of ^18^F-SU11248 was achieved by reacting the nitro precursor with ^18^F-fluoride using a nucleophilic substitution method. The radiochemical yield was reported to be 15–25%, with a radiochemical purity of greater than 95% after purification. These findings suggest that radiolabeling of ^18^F-SU11248 with its nitro precursor can be an efficient procedure for fluorine-18 incorporation.

Kniess et al. [79] synthesized and investigated the radiopharmacological properties of 3-[4′-[^18^F]fluorobenzylidene]indolin-2-one, a potential tyrosine kinase inhibitor, as a radiolabeled probe for PET imaging. The radiolabeling process was performed by nucleophilic substitution using ^18^F-fluoride, with a radiochemical yield of 48%. The radiochemical purity of the product was greater than 98% after purification. In vitro studies demonstrated that the compound exhibited good binding affinity to the target kinase (half-maximal inhibitory concentration (IC_50_) = 0.06–0.8 µM). The compound exhibited good in vitro stability. Furthermore, these results indicate that 3-[4′-[^18^F]fluorobenzylidene]indolin-2-one holds promise as a PET tracer for imaging tyrosine kinase activity in cancer; however, non-specific binding and off-target accumulation suggested the need for further structural modifications to enhance selectivity and imaging contrast.

Ilovich et al. [80] developed fluorine-18-labeled diaryl ureas as potential molecular imaging agents targeting VEGFR-2 and platelet-derived growth factor receptor (PDGFR), which are both involved in angiogenesis. The radiolabeling process was carried out using [^18^F]fluoride with a radiochemical yield of 46%. The synthesized compounds exhibited high radiochemical purity (99%) after purification. In vitro binding studies demonstrated strong affinity for both VEGFR-2 and PDGFR (IC_50_ = 5–15 nM), indicating the potential of these compounds as dual inhibitors for angiogenesis imaging.

Kuchar et al. [81] explored the potential of radioiodinated sunitinib as a radiotracer for imaging angiogenesis by developing 5-[^125^I]Iodo-sunitinib and evaluating its radiopharmacological properties. The radiosynthesis was achieved through electrophilic iodination, yielding radiochemical purities exceeding 98% and a radiochemical yield of approximately 95%. The logarithm of the distribution coefficient (logD) value of 5-[^125^I]Iodo-sunitinib was 2.25, indicating moderate lipophilicity, which is essential for membrane permeability and effective tumor uptake. In vitro stability assays confirmed the tracer’s stability in human plasma over 24 h, ensuring its viability for imaging applications. The IC_50_ values of 5-[^125^I]Iodo-sunitinib to inhibit cell proliferation were found to be 1.12 ± 0.07 and 1.81 ± 0.03 µM for HAEC and HT29 cells, respectively.

Biodistribution studies in mice demonstrated high thyroid uptake (~4 percent injected dose per gram of tissue (%ID/g) at 1 h post-injection (p.i.)), with sustained retention up to 4 h p.i. (~2.3 %ID/g), reflecting its binding affinity for VEGFR-2-associated angiogenesis sites. The tracer also exhibited hepatic metabolism, with moderate uptake in the liver (~8 %ID/g at 1 h p.i.), which decreased over time. Kidney accumulation was observed but was lower than typical small-molecule tracers, suggesting favorable clearance properties. These findings highlight 5-[^125^I]Iodo-sunitinib’s potential as an angiogenesis imaging agent, warranting further optimization and evaluation in PET-compatible isotopes such as ^124^I or ^18^F for clinical translation.

Hirata et al. [82] developed and evaluated novel radioiodinated anthranilate derivatives as potential SPECT tracers for imaging VEGFR expression in vivo. The synthesis involved electrophilic radioiodination, achieving high radiochemical purity (>95%) and radiochemical yields of approximately 60–70%. The lead compound exhibited a logP value of 4.2, indicating high lipophilicity. In vitro binding studies confirmed specific VEGFR-2 affinity, with inhibitory activity around 12.5 nM–2.53 μM, demonstrating strong potential as VEGFR imaging agents.

Biodistribution studies in tumor-bearing mice showed promising tumor uptake (~2.77 %ID/g at 30 min p.i.), with sustained retention up to 24 h (~0.85 %ID/g). The compounds exhibited moderate hepatic clearance (~3.93 %ID/g at 1 h), with renal excretion playing a significant role in tracer elimination. SPECT imaging studies confirmed clear tumor visualization, supporting the feasibility of these anthranilate derivatives for VEGFR-targeted imaging. These findings suggest that these radioiodinated anthranilates are promising candidates for noninvasive VEGFR imaging, potentially aiding in the assessment of angiogenesis in cancer and other diseases.

Poot et al. [77] investigated the radiosynthesis and preclinical evaluation of [^11^C]sorafenib as a PET tracer for imaging TKI in tumor-bearing mice. The synthesis was carried out via methylation of the phenolic hydroxyl group of sorafenib with [^11^C]methyl iodide, yielding a radiochemical purity of >99% and a radiochemical yield of approximately 50–60%. The tracer demonstrated high molar activity (150–210 GBq/μmol) and retained excellent in vitro stability (90–96%), remaining intact for 45 min in serum.

In vivo biodistribution studies in breast cancer and head and neck cancer mouse models revealed rapid tumor uptake of 1.09 %ID/g and 1.18 %ID/g at 5 min p.i., followed by an increase (2.11 %ID/g and 1.73 %ID/g at 1 h), respectively. The tracer showed specific binding (IC_50_ = 26 nM) to tumor tissues with high target-to-background ratios, indicating promising tumor-specific imaging potential. PET imaging confirmed good visualization of tumors, demonstrating the ability of [^11^C]sorafenib to monitor the expression and activity of targeted tyrosine kinases in vivo. These findings suggest that [^11^C]sorafenib is a potential PET tracer for evaluating tyrosine kinase inhibition in clinical oncology, especially for tumors sensitive to sorafenib treatment.

Samén et al. [83] developed the synthesis and preclinical evaluation of [^11^C]PAQ as a PET imaging tracer for VEGFR-2, which is implicated in angiogenesis. The radiosynthesis of [^11^C]PAQ was achieved by methylation of PAQ with [^11^C]methyl iodide, yielding a radiochemical purity of >99%. The radiochemical yield was 45–60% with a molar activity of approximately 40–50 GBq/µmol. The tracer demonstrated high stability in liver microsome solution, maintaining over 99% integrity at 90 min post-synthesis.

Preclinical evaluation in tumor-bearing mice showed promising in vivo characteristics. The tumor uptake of [^11^C]PAQ peaked at 8 %ID/g at 30 min p.i., followed by a rapid clearance from most tissues except for the tumors, where retention remained high. The target-to-background ratios improved with time, showing selective binding to VEGFR-2 expressed on tumor vasculature. PET imaging of tumor mice confirmed the specificity of the tracer for VEGFR-2 expression. These findings suggest that [^11^C]PAQ is a viable PET tracer for non-invasive imaging of VEGFR-2 expression, which could potentially aid in evaluating angiogenesis in cancer and assessing the efficacy of anti-angiogenic therapies.

Gao et al. [84] investigated the radiosynthesis of two [^11^C]labeled derivatives of vandetanib-[^11^C]vandetanib and [^11^C]chloro-vandetanib as potential PET agents for imaging VEGFR in cancer. The radiochemical purity of both compounds was >99%, and the radiochemical yield was 40–50%. The synthesis was achieved by reacting [^11^C]methyl iodide with the appropriate precursor compounds, resulting in high molar activities of 370–555 GBq/µmol for both tracers. The tracers demonstrated selective binding to VEGFR-expressing cells with IC_50_ values of 40, 110, and 500 nM for VEGFR-2, VEGFR-3, and EGFR, respectively. These results suggest that both tracers are promising candidates for PET imaging of VEGFR in diseases, offering a non-invasive means to monitor angiogenesis and assess the effectiveness of anti-VEGF therapies.

Despite their advantages, it should be noted that small molecules face challenges such as rapid clearance, non-specific binding, and metabolic instability, which can hinder their effectiveness in imaging applications. However, in VEGFR-targeted imaging, their ability to cross the BBB facilitates efficient visualization of brain tumors. Moreover, their dual functionality enables their use as both imaging agents and therapeutic drugs, enhancing their versatility in cancer diagnostics and treatment [85].

#### 1.2.2. Radiolabeled Peptides

Peptides are multifunctional elements that are abundant in living systems and involved in many biological functions. Due to their high selectivity and ability to target, new diagnostic and therapeutic techniques have been developed by radiolabeling these peptides [86].

Kang et al. [86] explored the preclinical efficacy of a radiolabeled peptide, ^68^Ga-NOTA-VEGF-121, for imaging VEGFR-2 expression in tumor-bearing mice using microPET. VEGFR-2 is a key angiogenic marker associated with tumor growth, and VEGF-121 is a ligand targeting VEGFR-2. The chelating agent 1,4,7-triazacyclononane-1,4,7-triacetic acid (NOTA) was used to radiolabel VEGF-121 with ^68^Ga eluted from a ^68^Ge/^68^Ga generator, yielding a radiochemical efficiency of 40 ± 4.5% and a molar activity of 243.1 ± 104.6 GBq/μmol. After labeling, ^68^Ga-NOTA-VEGF-121 displayed a high serum stability (97% at 4 h) and a binding affinity to VEGFR-2 comparable to VEGF-121 alone, indicating that NOTA conjugation did not compromise the affinity.

In vitro studies showed that human aortic endothelial cells (HAECs) took up ^68^Ga-NOTA-VEGF-121 in a time-dependent manner, reaching 11%ID at 4 h. Blocking experiments with a VEGFR-2 antibody reduced the uptake by 40%, confirming specific VEGFR-2-mediated uptake. In vivo, microPET region-of-interest analysis in U87MG tumor-bearing mice showed increased tumor uptake over time, with 2.97 ± 0.06 %ID/g at 4 h. The tumor-to-muscle uptake ratio increased from 2.9 at 1 h to 3.1 at 4 h.

Biodistribution studies showed the highest accumulation in the liver (40.90 ± 4.20 %ID/g) and spleen (21.15 ± 1.56 %ID/g), with moderate tumor uptake (1.46 ± 0.20 %ID/g at 1h to 1.90 ± 0.02 %ID/g at 4 h) and tumor-to-muscle ratios of 2.9 at 1 h, 3.2 at 2 h, and 3.1 at 4 h. Immunofluorescence staining of U87MG tumor sections demonstrated pronounced co-localization of VEGFR-2 with the endothelial cell marker CD31, confirming that VEGF receptors are predominantly localized on tumor vasculature. The overlap of VEGFR-2 and CD31 signals in the tumor microenvironment highlights that VEGF receptor expression is mainly associated with endothelial cells rather than tumor parenchymal cells. This spatial correlation supports the specificity of ^68^Ga-NOTA-VEGF121 observed in PET imaging, where significant tracer accumulation was detected within the tumor region, reflecting high VEGFR expression on tumor blood vessels. Thus, the immunofluorescence data, through direct comparison with CD31, validate the PET imaging results by confirming that ^68^Ga-NOTA-VEGF121 uptake corresponds to angiogenic endothelial structures, reinforcing its utility as a non-invasive biomarker for tumor angiogenesis. Although the liver and spleen uptakes were similar to those of ^68^Ga-NOTA-benzyl-VEGF-121, the tumor uptake was significantly improved (*p* < 0.01) with ^68^Ga-NOTA-VEGF-121. This radiotracer effectively demonstrated specific VEGFR-2 targeting in vivo, marking it as a promising imaging agent for monitoring tumor angiogenesis and VEGFR expression.

Hu et al. [10] developed a first-of-its-kind peptide-based PET tracer targeting VEGFRs, which are key biomarkers of tumor angiogenesis. The researchers synthesized a novel tracer, ^64^Cu-VEGF_125–136_, by labeling a 12-amino-acid peptide (VEGF_125–136_) with the radioisotope copper-64 using a DOTA chelator and a polyethylene glycol (PEG) spacer to enhance solubility and reduce steric hindrance. The radiolabeling process achieved a high radiochemical yield (>95%) and purity (>98%), with a molar activity of 74.3 ± 3.8 GBq/μmol, indicating suitability for clinical translation.

In vivo studies demonstrated that ^64^Cu-VEGF_125–136_ exhibited high tumor uptake, notably 5.89 %ID/g at 20 min p.i. in B16F10 melanoma-bearing mice and provided excellent imaging quality within 1 h. The tracer’s uptake correlated strongly with VEGFR expression levels across different tumor models, as confirmed by immunofluorescence staining. Blocking studies using unlabeled peptides resulted in over 90% reduction in tumor uptake, confirming specificity. Additionally, the tracer showed favorable pharmacokinetics, characterized by rapid blood clearance (half-life ~20 min) and minimal nonspecific organ accumulation, which enhanced image contrast and reduced background noise. These findings suggest that ^64^Cu-VEGF_125–136_ is a promising PET imaging agent for noninvasive visualization of VEGFR expression, with potential applications in cancer diagnosis, treatment monitoring, and therapeutic development.

Hao et al. [87] discussed the potential of the dimeric peptoid GU40C4, a potent VEGFR-2 antagonist that inhibits angiogenesis and tumor growth, as a PET imaging agent for VEGFR-2 expression. GU40C4 and a control peptoid were modified by introducing a cysteine residue at the C-terminus, facilitating conjugation with a bifunctional chelator (DOTA) via maleimide-thiol coupling chemistry. The DOTA-conjugated peptoids were labeled with the positron emitter ^64^Cu (half-life 12.7 h) with high radiochemical yields (>90%) and molar activity ranging from 10–80 GBq/μmol. DOTA conjugation had minimal impact on the VEGFR-2 binding affinity of GU40C4 (GU40C4, IC_50_ = 756 ± 14 nM; DOTA-GU40C4, IC_50_ = 623 ± 98 nM; *p* = 0.25), maintaining its functionality as a VEGFR-2 antagonist.

PET imaging in a prostate cancer xenograft (PC3) mouse model demonstrated significant and sustained accumulation of ^64^Cu-DOTA-GU40C4 in VEGFR-2-positive PC3 tumors at 1, 4, and 20 h p.i. (2.25 ± 0.24, 2.15 ± 0.34, and 1.90 ± 0.18 %ID/g, respectively). The tumor uptake was significantly higher than that observed for the control peptoid conjugate (0.3–0.5 %ID/g; *p* < 0.001 at all time points).

Radioactivity accumulation in mouse salivary glands was also observed (3.17 ± 0.25, 3.00 ± 0.36, and 1.83 ± 0.21 %ID/g at 1, 4, and 20 h, respectively), with VEGFR-2 expression confirmed by polymerase chain reaction (PCR) analysis. The marked difference in tumor and salivary gland uptake between ^64^Cu-DOTA-GU40C4 and the control peptoid highlights the specificity of GU40C4 for VEGFR-2. The uptake of ^64^Cu-DOTA-GU40C4 in the heart and lungs was moderate, ranging from approximately 1.3 to 2.0 %ID/g, compared to the low uptake observed in the control group, which ranged from about 0.3 to 0.6 %ID/g. In contrast, the liver showed high uptake in both groups with ^64^Cu-DOTA-GU40C4 (~24–18 %ID/g) higher than that of the control group (~13–12 %ID/g) during the 20 h p.i.

The study demonstrates ^64^Cu-DOTA-GU40C4 as a viable PET imaging agent for VEGFR-2 expression. The selective accumulation of the radiotracer in VEGFR-2-positive tissues underscores its potential for assessing tumor vascular activity and guiding anti-angiogenic therapy. Furthermore, the unexpected visualization of VEGFR-2 expression in the salivary glands introduces new avenues for exploring VEGFR-2’s physiological roles.

Masłowska et al. [88] synthesized two radiolabeled inhibitors targeting the VEGF-A_165_/NRP-1 complex, a key player in tumor angiogenesis. The researchers developed radioconjugates based on the A7R peptide and a branched peptidomimetic, Lys(hArg)-Dab-Pro-Arg, each linked via a 6-aminohexanoic acid (Ahx) spacer to a DOTA chelator. These constructs were labeled with gallium-68 for diagnostic imaging and lutetium-177 for potential therapeutic applications. The radiolabeling efficiency exceeded 95% for both isotopes. In vitro stability assessments in phosphate-buffered saline (PBS) and cysteine/histidine solutions showed that ^68^Ga-labeled compounds maintained over 90% integrity for at least 3 h, while ^177^Lu-labeled counterparts remained stable for at least 6 days. However, both radioconjugates exhibited significant degradation in human serum, with less than 50% remaining intact after 1 h, indicating susceptibility to enzymatic breakdown. Despite this, enzyme-linked immunosorbent assay (ELISA) demonstrated that the branched peptidomimetic had a lower IC_50_ value (IC_50_ = 0.2 µM) for NRP-1 compared to A7R (IC_50_ = 5.9 µM), suggesting its potential as a more effective inhibitor.

The study concludes that while the synthesized radioconjugates show promise as theranostic agents targeting NRP-1, their rapid degradation in human serum limits their immediate clinical applicability. The authors suggest that further structural modifications are necessary to enhance serum stability without compromising binding affinity. These findings contribute to the ongoing development of targeted radiopharmaceuticals for cancer diagnosis and therapy, emphasizing the need for balancing molecular stability with biological efficacy.

While peptides offer versatility, rapid distribution, and non-immunogenicity in developing novel VEGFR imaging agents, their application faces challenges, including susceptibility to enzymatic degradation and their short biological half-life. Nonetheless, research is still emerging to develop peptide-based imaging agents with high VEGFR specificity, increased stability, and optimized pharmacokinetics.

#### 1.2.3. Radiolabeled Antibodies

Antibodies are highly specialized proteins that play a pivotal role in the immune system by recognizing and binding to specific antigens with exceptional affinity and specificity. This high degree of specificity makes them ideal candidates for targeting biomolecules or cells, such as those overexpressed in cancer or other diseases. Additionally, antibodies typically have relatively long circulation times in the bloodstream, allowing them to reach and accumulate at target sites efficiently [89]. These properties make antibodies particularly suitable for radiolabeling, as they can deliver radionuclides directly to the desired biological targets while minimizing off-target effects. This targeted delivery enhances the efficacy and safety of diagnostic imaging and therapeutic applications, making antibodies a cornerstone of modern radiopharmaceutical development [90].

Nagengast et al. [69] radiolabeled bevacizumab, an antibody targeting VEGF-A, with isotopes ^89^Zr and ^111^In for imaging applications. The labeling methods included ^89^Zr conjugation via N-succinyldesferrioxamine B-tetrafluorophenol (N-sucDf-TFP) and ^111^In labeling through 2-(4-isothiocyanatobenzyl)-diethylenetriaminepentaacetic acid (ITC-DTPA). The labeling yields were high for both radiotracers (98.0 ± 0.7% for ^89^Zr-bevacizumab and 96.6 ± 0.5% for ^111^In-bevacizumab), with high stability in human serum at 37 °C after 168 h, indicating minimal radiochemical degradation (6%). ^89^Zr-beavcizumab showed 54.0 ± 3.7% binding, while ^111^ln-bevacizumab had 56.9 ± 0.7% binding compared with nonradiolabeled bevacizumab. The biodistribution study showed that the tumor uptake for ^89^Zr-bevacizumab was higher (6.82 ± 1.80 %ID/g) when compared with that of control ^89^Zr-IgG (2.87 ± 0.48 %ID/g) at 168 h p.i.. Moreover, except for the kidney, where ex vivo biodistribution showed higher accumulation in the control (^89^Zr-IgG), all other organs showed an equal distribution pattern for ^89^Zr-bevacizumab and ^89^Zr-IgG. The ex vivo biodistribution results were similar for ^89^Zr-bevacizumab and ^111^In-beavcizumab at 24, 72, and 168 h p.i.. Overall, the tumor uptake with ^111^In-bevacizumab and ^89^Zr-bevacizumab was significantly higher than that of the control. The results showed that there was a positive correlation between the tracer uptake and VEGF-A expression levels, which demonstrates that this method can be used to assess the response to anti-angiogenic therapy. However, concerns were noted regarding the radiation exposure from ^89^Zr due to its high-energy gamma emissions and limited data on in vivo quantification for ^111^In-bevacizumab [69].

Csikos et al. [91] investigated the PET/MRI imaging of a cervical carcinoma mouse model with ^52^Mn-labeled bevacizumab (targeting VEGF-A). Bevacizumab was conjugated with the chelator p-NCS-Bn-DOTA-GA to form ^52^Mn-DOTAGA-bevacizumab. The radiolabeling resulted in a high radiochemical yield (>90%), and the tracer demonstrated excellent stability in both EDTA and oxalic acid solutions, with minimal loss of radiochemical integrity (~5%) over 48 h. In mouse serum, the tracer retained >70% for up to 7 days, underscoring its stability.

Biodistribution and PET imaging showed initial accumulation of ^52^Mn in organs such as the liver, kidney, pancreas, and salivary glands, with reduced liver and kidney signal over time, though pancreas and salivary gland retention persisted. Notably, PET imaging enabled clear visualization of KB-3-1 cervix tumors as early as 4 h p.i., with increasing tracer uptake in tumors observed over time, indicating effective tumor localization.

Immunohistochemical analysis confirmed target specificity, showing strong VEGF expression on cancer cell membranes 10 days p.i.. This study demonstrates that ^52^Mn-DOTAGA-bevacizumab can effectively localize tumor tissues with low off-target uptake, highlighting its potential for VEGF-A-targeted imaging in oncology [91].

Van Es et al. [92] used ^89^Zr-labeled bevacizumab (targeting VEGF-A) to assess tumor burden and treatment response in patients with metastatic renal cell carcinoma (mRCC). Bevacizumab was conjugated with tetrafluorphenol-N-succinyldesferal-Fe, and ^89^Zr radiolabeling was conducted under Good Manufacturing Practice (GMP) conditions, yielding a radiochemical purity of over 95%. PET/CT imaging was performed to visualize and quantify ^89^Zr-bevacizumab uptake in tumor lesions and healthy organs.

Of 147 CT-detected tumor lesions larger than 10 mm, 71% were identifiable on PET. Maximum standardized uptake value (SUVmax) analysis of 94 quantifiable lesions revealed significant variability between patients, with an eight-fold range in baseline SUVmax values. After two weeks of everolimus treatment, a median SUVmax decrease of 9.1% was observed (*p* < 0.0001), and in six weeks, this was further decreased to 23.4% in 70 lesions, indicating a reduction in ^89^Zr-bevacizumab uptake. All patients continuing treatment achieved stable disease status at three months. An exploratory analysis showed a significant correlation (r = 0.63, *p* = 0.02) between baseline SUVmax and time on treatment, though changes in SUVmax did not predict time on treatment.

This study concludes that ^89^Zr-bevacizumab PET can effectively visualize mRCC lesions and monitor response to everolimus treatment. However, the utility of ^89^Zr-bevacizumab PET for early progression detection remains undetermined, and larger trials are needed to validate its role in patient selection for VEGF-targeted therapies.

Paudyal et al. [93] examined the application of PET imaging using ^64^Cu-labeled bevacizumab to target VEGF in colorectal cancer (CRC) models. This study addressed the need for advanced imaging to monitor VEGF expression in vivo, as VEGF is a critical promoter of angiogenesis in CRC and other solid tumors. The authors used ^64^Cu-labeled bevacizumab for PET imaging in CRC xenografts. The radiolabeling process was carefully controlled to preserve the antibody’s specificity for VEGF while enabling high-quality imaging. Human colorectal cancer cell lines were implanted in immunocompromised mice to create xenograft models that recapitulate human tumor growth and VEGF expression in vivo. PET scans were conducted at various intervals post-injection of ^64^Cu-labeled bevacizumab to evaluate the biodistribution and VEGF targeting over time. These intervals allowed the researchers to observe the antibody’s uptake and retention in the tumor tissue versus normal tissues.

The PET images revealed a high uptake of ^64^Cu-labeled bevacizumab in the tumor tissue, which peaked at 24 h p.i. compared with non-tumor regions. The high tumor uptake was further confirmed by a biodistribution study. This selective accumulation indicates that the labeled antibody effectively targets VEGF expressed in the CRC xenografts, confirming the utility of ^64^Cu-bevacizumab as a VEGF-specific imaging agent.

Quantitative PET analysis showed that ^64^Cu-bevacizumab had prolonged retention in tumors, with relatively low uptake in non-target organs, except for some accumulation in the liver due to the metabolism of radiolabeled antibodies. These findings suggest that the tracer is effective for sustained imaging of VEGF over extended periods. This method may predict which tumors are likely to respond to anti-VEGF therapies like bevacizumab, facilitating personalized treatment approaches. However, the authors note that while ^64^Cu provides good imaging properties and a half-life compatible with antibody biodistribution, there may be challenges with liver uptake affecting signal clarity. Future improvements in imaging probe design could minimize non-specific uptake and enhance tumor contrast.

Oosterwijk-Wakka et al. [94] evaluated the efficacy of combining sunitinib, a VEGFR-targeting tyrosine kinase inhibitor, with ^177^Lu-labeled cG250 radioimmunotherapy (RIT) in treating advanced renal cell carcinoma (RCC). The study utilized two human RCC xenograft models: NU12 (sunitinib-sensitive) and SK-RC-52 (sunitinib-resistant), both expressing carbonic anhydrase IX (CAIX), the target of cG250. Mice were treated with sunitinib alone (40 mg/kg/day), ^177^Lu-cG250 RIT alone (6.5 MBq/10 µg ^177^Lu-cG250), or a combination. Tumor volume, vascular permeability, and resistance marker expression were analyzed to determine treatment efficacy.

In the SK-RC-52 model, neither a single cycle of sunitinib (*p* = 0.168, day 14) nor ^177^Lu-cG250 alone (*p* = 0.126, day 48) produced significant tumor growth delay. In contrast, the combination of sunitinib and ^177^Lu-cG250 RIT resulted in a substantial and statistically significant tumor growth delay (*p* < 0.001, day 48). Notably, two cycles of the combination therapy induced near-complete tumor stasis (*p* < 0.001), with survival rates of 91% and 85% following two and one cycles, respectively, superior to ^177^Lu-cG250 RIT alone (69% and 54% survival). Histological analyses confirmed complete tumor eradication in several mice, with no viable tumor cells detected.

In the NU12 model, treatment began earlier (day 17 post-inoculation), and both monotherapies and combination therapies produced immediate tumor growth delays. However, while single-cycle treatments allowed for eventual tumor regrowth (∼350 mm^3^ by day 42 in sunitinib-only mice), two cycles of ^177^Lu-cG250 RIT or sunitinib plus ^177^Lu-cG250 RIT achieved sustained complete tumor regression. By day 147, all mice in these groups survived, and over 80% remained tumor-free. Histopathology confirmed cure in 83% (sunitinib plus RIT) and 86% (RIT-only) of animals. By comparison, single-cycle ^177^Lu-cG250 RIT cured only 29% of mice, and monotherapy with sunitinib resulted in far fewer cures (8–14%). These findings strongly support the therapeutic synergy of sequential sunitinib and RIT in targeting both the vascular and cellular components of advanced RCC. Immunohistochemical analysis revealed elevated expression of phosphorylated AXL receptor tyrosine kinase (AXL) and mesenchymal epithelial transition receptor tyrosine kinase (MET) in SK-RC-52 tumors, suggesting activation of resistance pathways. These findings highlight the potential of combining anti-angiogenic therapy with targeted RIT to overcome drug resistance in RCC.

Mitran et al. [95] highlighted the potential of a novel biparatopic affibody-based radiopharmaceutical for non-invasive imaging of VEGFR-2 expression in glioma vasculature. This innovative affibody construct was specifically designed to bind two distinct, non-overlapping epitopes on VEGFR-2, a strategy that significantly enhanced binding affinity, specificity, and tumor retention compared with traditional monovalent constructs.

In this study, a biparatopic affibody was engineered to optimize receptor engagement and improve binding specificity. The biparatopic affibody was conjugated to 1,4,7-triazacyclononane-1-glutamic acid-4,7-diacetic acid (NODAGA), a chelator compatible with indium-111 (^111^In), a single photon-emitting radionuclide. The radiolabeling process achieved high radiochemical purity (>95%) and molar activity, making it suitable for in vivo imaging. In vitro studies using VEGFR-2-expressing cell lines confirmed the affibody’s high binding specificity and affinity. Competitive binding assays further validated the biparatopic design, demonstrating superior receptor interaction and tumor retention compared with monovalent constructs.

The preclinical evaluation was conducted using a murine model with subcutaneous glioma xenografts. SPECT imaging following intravenous injection of the radiolabeled affibody showed significant accumulation in glioma tumors, effectively visualizing VEGFR-2 expression in the tumor vasculature. High tumor-to-blood and tumor-to-muscle ratios of 11 and 15, respectively, were observed at 2 h p.i.; while the imaging agent rapidly cleared from the non-target tissues, particularly the liver and kidneys. Blocking studies, where non-radiolabeled affibody was co-administered, significantly reduced tumor uptake, confirming VEGFR-2-specific targeting. The in vivo study further demonstrated that the biparatopic affibody outperformed monovalent constructs in imaging performance by enhancing receptor engagement and retention in tumors. The findings show the potential of biparatopic affibody-based radiopharmaceuticals for VEGFR-2-targeted molecular imaging, particularly in glioma. The innovative approach provides a strong foundation for further development toward clinical translation, improving the diagnosis and management of VEGFR-2-associated malignancies.

Despite their advantages in VEGFR imaging, antibodies have certain limitations. Their large size can slow clearance from non-target tissues, resulting in elevated background signals, reduced image contrast, and increased radiation exposure, particularly at early time points following injection [96]. New research aims to address these challenges by modifying the antibodies to improve tissue penetration and faster clearance from the body. Small molecular monobodies may retain the binding and shorten the circulation time. With these new strategies, antibodies can be engineered to improve pharmacokinetics for VEGFR imaging [97].

Biomolecules, such as monobodies and nanobodies, offer distinct advantages and trade-offs when compared with traditional antibodies in therapeutic and diagnostic applications. Monobodies, engineered protein scaffolds derived from fibronectin or other non-antibody frameworks, and nanobodies, derived from the single-domain antibodies of camelids, are significantly smaller than full-length antibodies [98]. This small size enhances their tissue penetration, enabling better access to targets in dense or poorly vascularized tissues, such as tumors. Additionally, monobodies and nanobodies lack the Fc region of traditional antibodies, reducing off-target immune activation and enhancing safety in certain applications. Their rapid renal clearance can minimize background signal in imaging but may necessitate modifications, such as PEGylation, to extend their half-life for therapeutic use [99,100]. Compared with small molecules, monobodies and nanobodies retain high specificity for their targets, combining the precision of antibodies with the versatility of compact agents. However, their smaller size may result in reduced binding avidity compared with full-length antibodies, which rely on bivalent binding for enhanced stability. Overall, these small biomolecules bridge the gap between small molecules and traditional antibodies, offering unique opportunities in precision medicine, especially for applications requiring rapid clearance or deep-tissue targeting [101].

Tan et al. [102] explore the use of a radiolabeled anti-VEGF-A antibody for non-invasive imaging of atherosclerotic plaque neovascularization and assessment of atorvastatin’s antiangiogenic effects. Researchers developed a SPECT imaging agent, ^99m^Tc-MAG3-bevacizumab, which targets VEGF-A—a key mediator of angiogenesis implicated in plaque vulnerability. Using ApoE−/− mice, a model prone to atherosclerosis, the study demonstrated that this tracer could effectively visualize neovascularization within plaques. In vivo micro-SPECT/CT and ex vivo BSGI planar imaging revealed higher tracer uptake in the aortas of untreated ApoE−/− mice compared to those treated with atorvastatin or wild-type controls, indicating the tracer’s specificity for neovessels.

Histological analyses corroborated imaging findings. Oil Red O staining showed a significant reduction in lipid-rich plaque areas in atorvastatin-treated mice (22.67 ± 4.53%) compared to untreated ApoE−/− mice (43.24  ±  6.13%; *p*  =  0.0017). Immunohistochemistry for CD31 and VEGF indicated decreased neovascularization in treated mice, aligning with the reduced tracer uptake observed in imaging studies. These results demonstrate that ^99m^Tc-MAG3-bevacizumab SPECT imaging can be a promising tool for detecting plaque neovascularization and monitoring the efficacy of antiangiogenic therapies, such as atorvastatin, in atherosclerosis.

Lee et al. [103] developed chitosan-DC101 conjugates for targeted molecular imaging of VEGF receptors overexpressed in ischemic microvasculature. The radiolabeling of DC101 with ^99m^Tc was carried out with a high radiolabeling efficiency of over 95%, confirmed by ITLC and HPLC. The yield of the radiolabeling process was approximately 80%. In vivo biodistribution studies conducted in mice revealed that the radiolabeled chitosan-DC101 conjugates demonstrated specific targeting of VEGF receptors in ischemic tissues (2.39 %ID/g and 3.03 %ID/g at 12 and 24 h p.i., respectively). The study also highlighted the rapid blood clearance and minimal nonspecific accumulation in normal tissues, indicating the potential of the chitosan-DC101 conjugates for imaging angiogenesis in ischemic regions. These findings suggest that the conjugates can be effective for in vivo imaging of ischemic microvasculature with a high level of specificity.

Camacho et al. [104] synthesized ^99m^Tc(CO)_3_-radiolabeled bevacizumab for molecular imaging of melanoma. The radiolabeling achieved a high radiochemical purity of 98%, confirmed by ITLC and HPLC analysis. The yield of the labeling reaction was around 97% at 37 °C. In vivo biodistribution studies in melanoma-bearing mice demonstrated significant tumor uptake (2.64 %ID/g at 4 h p.i.), showing that ^99m^Tc(CO)_3_-bevacizumab specifically targeted VEGF receptors in melanoma tissues. The conjugate showed rapid blood clearance and minimal non-specific accumulation in other organs, indicating its potential for targeted imaging of melanoma.

In the follow-up study, Camacho et al. [105] explored a different approach via HYNIC for radiolabeling bevacizumab with ^99m^Tc in over 90% radiochemical purity and yield. In vivo imaging studies in melanoma models showed good tumor uptake, with a value of 5.49% ID/g at 4 h p.i.. The conjugate demonstrated high specificity for tumor cells and excellent tumor-to-background contrast, making it a promising candidate for non-invasive imaging of melanoma with potential for clinical translation. Both studies highlight the effectiveness of ^99m^Tc-labeled bevacizumab in targeting melanoma through VEGF receptor imaging.

Kameswaran et al. [106] synthesized ^99m^Tc(CO)_3_-DTPA-bevacizumab for targeted molecular imaging in a melanoma model. The radiolabeling achieved a high radiochemical purity of 98%. In vivo biodistribution studies in melanoma-bearing mice revealed significant tumor uptake of the radiolabeled bevacizumab (6.9 %ID/g at 24 h p.i.). The conjugate demonstrated specific targeting of VEGF receptors on melanoma cells, with minimal non-specific accumulation in other tissues. Rapid blood clearance and favorable tumor-to-background ratios suggested that ^99m^Tc(CO)_3_-DTPA-bevacizumab could be an effective radiopharmaceutical for non-invasive imaging of melanoma with high specificity and promising clinical potential.

Janousek et al. [107] investigated the radiolabeling of the antiangiogenic human monoclonal antibody ramucirumab with ^99m^Tc for imaging VEGFR-2-positive cell lines. The radiolabeling process achieved over 95% radiochemical purity. The study also highlighted the stability of the radiolabeled conjugate, with minimal decomposition of radiotracer over 24 h. These findings suggest that ^99m^Tc-ramucirumab could serve as a promising candidate for non-invasive molecular imaging of VEGFR-2 expression in cancer, offering a potential diagnostic tool for antiangiogenic therapies.

Zhang et al. [108] developed a dual-labeled bevacizumab conjugate for PET and near-infrared fluorescence (NIRF) imaging of vascular endothelial growth factor (VEGF). Bevacizumab was labeled with both ^64^Cu and IRDye 800CW (a near-infrared fluorophore) to enable multimodal imaging. The radiochemical yield of ^64^Cu labeling was above 80%, with a radiochemical purity of >98%. The immunoreactivity of the dual-labeled antibody remained high, ensuring VEGF specificity. In vivo PET imaging in xenograft mouse models showed high tumor uptake 4.6 ± 0.7, 16.3 ± 1.6, 18.1 ± 1.4, and 20.7 ± 3.7 %ID/g at 4, 24, 48, and 72 h p.i., respectively, with a favorable tumor-to-muscle contrast. NIRF imaging confirmed strong fluorescence signals in tumors, correlating with PET data. The study demonstrated the feasibility of dual-labeled bevacizumab for noninvasive, high-sensitivity imaging of VEGF expression in tumors, supporting its potential use in cancer diagnosis and therapy monitoring.

Luo et al. [109] investigated ⁶⁴Cu-labeled ramucirumab as a PET tracer for imaging VEGFR-2 expression in lung cancer. The immunoreactivity of the labeled antibody was preserved (>85%), ensuring specific VEGFR-2 targeting. In vivo PET imaging in lung cancer xenografts (HCC4006 and A549 models) showed high tumor uptake (~9.5 ± 2.2 %ID/g at 48 h p.i. in HCC4006 and 2.2 ± 0.7 %ID/g in A549), correlating with VEGFR-2 expression levels. Tumor-to-muscle ratios were favorable (~9:1 in HCC4006 and ~4:1 in A549), confirming specificity. Biodistribution studies revealed primary clearance through the liver. This study demonstrated the potential of ⁶⁴Cu-NOTA-ramucirumab for noninvasive imaging of VEGFR-2 in lung cancer, supporting its application in tumor characterization and treatment monitoring.

Gaykema et al. [110] evaluated ^89^Zr-labeled bevacizumab for PET imaging in primary breast cancer to assess VEGF-A expression. ^89^Zr-bevacizumab was synthesized with a radiochemical purity >95%. PET imaging in 23 breast cancer patients showed tumor uptake of SUV_max_ (1.85 ± 1.22 in breast tumors versus 0.59 ± 0.37 in ipsilateral normal breast tissue). Immunohistochemistry confirmed VEGF-A expression in tumors that correlated directly with tracer uptake. The study demonstrated that ^89^Zr-bevacizumab PET imaging is feasible in breast cancer, but further research is needed to establish its clinical utility in patient stratification and therapy monitoring.

Bahce et al. [111] conducted a pilot study using ^89^Zr-bevacizumab PET imaging in patients with advanced non-small cell lung cancer (NSCLC) to evaluate VEGF-A expression. ^89^Zr-bevacizumab was prepared with a radiochemical purity >97% and administered at 37 MBq (5 mg bevacizumab) per patient. PET scans were performed four and seven days p.i.. High liver uptake was observed due to antibody clearance. Immunohistochemical VEGF-A staining correlated with the PET signal in some cases but not all. This study demonstrated the feasibility of ^89^Zr-bevacizumab PET for visualizing VEGF-A expression in NSCLC, although further validation is required for clinical decision-making.

Cohen et al. [112] explored the inert coupling of IRDye800CW to monoclonal antibodies along with ^89^Zr incorporation for clinical optical imaging of tumor targets. ^89^Zr-labeling was used to accurately quantify the biodistribution of the antibodies labeled with various amounts of IRDye800CW. The conjugation process achieved radiochemical purity greater than 95% with high yields of 75%. The conjugates exhibited strong optical properties and retained the antibody binding affinity essential for targeting tumor cells. The study demonstrated that these IRDye800CW-labeled antibodies could be used for fluorescence-guided surgery in preclinical tumor models, showing excellent tumor-targeting efficacy. The clearance of the conjugates from non-target tissues was rapid, ensuring a favorable tumor-to-background ratio. These findings support the potential of IRDye800CW conjugates in clinical optical imaging, especially for real-time tumor visualization during surgery [112,113].

Li et al. [114] investigated the use of ^89^Zr-labeled ramucirumab for immuno-PET imaging of VEGFR-2 expression in prostate cancer. The radiolabeling process using ^89^Zr-desferrioxamine (DFO)-ramucirumab achieved a radiochemical yield of ~80% with high radiochemical purity (>98%). In vitro assays demonstrated high VEGFR-2 affinity in prostate cancer cells. In vivo PET imaging in prostate cancer xenografts showed significant tumor uptake, with values of 6.6 ± 1.7 %ID/g at 24 h p.i., peaking at 9.6 ± 2.3 %ID/g at 96 h p.i., followed by gradual clearance. The tumor-to-muscle ratio at 48 h was ~7:1, confirming the tracer’s specificity and strong imaging contrast.

Biodistribution studies indicated primary clearance via the hepatic system, with moderate liver uptake (~5.4 %ID/g at 48 h p.i.), consistent with antibody metabolism. Minimal uptake was observed in non-target tissues, supporting a favorable imaging profile. PET imaging results correlated with VEGFR-2 expression levels, confirming the potential of ^89^Zr-ramucirumab for noninvasive assessment of VEGFR-2 in prostate cancer. This study demonstrated that ^89^Zr-ramucirumab immuno-PET could be a valuable tool for tumor characterization, patient stratification, and monitoring of anti-angiogenic therapies in clinical oncology.

Kameswaran et al. [115] evaluated ^177^Lu-labeled CHX-A″-DTPA-bevacizumab for RIT of VEGF-expressing cancers. The conjugation of CHX-A″-DTPA to bevacizumab was confirmed via mass spectrometry, and subsequent ^177^Lu labeling achieved a radiochemical purity of >98% after purification. The complex exhibited high stability in serum for up to 4 days, with minimal transchelation or degradation, supporting its suitability for in vivo applications.

Biodistribution studies in tumor-bearing mice demonstrated high tumor uptake (21.8 ± 2.8 %ID/g at 24 h p.i.), which persisted over time due to the long circulation half-life of the antibody. Uptake in organs involved in monoclonal antibody metabolism, such as the liver (13.7 ± 0.6 %ID/g) and spleen (6.7 ± 0.2 %ID/g), was also observed. The tumor-to-blood ratio increased over time, suggesting effective clearance from non-target tissues. These findings indicate that ^177^Lu-CHX-A″-DTPA-bevacizumab is a promising agent for targeted RIT of VEGF-expressing tumors, warranting further preclinical and clinical investigations.

Rainer et al. [116] investigated the prognostic value of ^123^I-VEGF scintigraphy in glioma patients, aiming to non-invasively assess tumor angiogenesis. The study included 23 patients (mean age 56.6 ± 14.4 years) with histologically confirmed gliomas of varying World Health Organization (WHO) grades. Each patient underwent SPECT imaging at 30 min and 18 h p.i. of ^123^I-VEGF. Additionally, a subset of eight patients also received ^11^C-methionine positron emission tomography ([^11^C]-MET PET) to compare metabolic and angiogenic imaging.

The results demonstrated that ^123^I-VEGF uptake was highly specific to WHO grade IV gliomas: 14 of 16 patients with grade IV tumors showed significant uptake at 18 h, whereas no increased uptake was observed in patients with grade II or III tumors. The tumor-to-normal brain (T/N) uptake ratio of ^123^I-VEGF served as a critical prognostic indicator. A T/N threshold of 1.32 was established: patients below this threshold had a significantly longer mean overall survival (OS) of 2680 days, compared to only 295 days for those above the threshold (*p* = 0.002). Even within the high-grade glioma group, a higher cutoff of 1.75 further stratified the prognosis, with an OS of 720 days versus 183 days (*p* < 0.05). For comparison, [^11^C]-MET PET demonstrated a mean T/N ratio of 3.71 in grade IV gliomas versus 1.74 in lower-grade tumors, underscoring its utility in tumor grading. However, ^123^I-VEGF added value by offering survival-related angiogenic insight not captured by metabolic imaging alone. These findings suggest that ^123^I-VEGF imaging could be a powerful tool in both glioma classification and outcome prediction.

Collingridge et al. [117] developed ^124^I-labeled VG76e, a radiolabeled monoclonal antibody targeting VEGF, for in vivo PET imaging of tumor angiogenesis. The radiolabeling was achieved via direct iodination using ^124^I, yielding a radiochemical purity >94% and a radiochemical yield of ~16.3 ± 8.7%. In vitro binding assays confirmed its specificity for VEGF, making it a promising candidate for imaging VEGF expression in tumors.

In biodistribution studies using a human tumor xenograft model, tumor uptake was quantified at 5 min, 1 h, 24 h, and 48 h p.i.. The results showed high uptake at 24 and 48 h for HT1080-26.6 and HT1080-1/3C tumor types, respectively. Background uptake was observed in organs involved in antibody metabolism, such as the liver and spleen. PET imaging confirmed high tumor-to-muscle contrast, supporting the feasibility of ^124^I-VG76e for noninvasive VEGF imaging. These findings highlight its potential for monitoring anti-angiogenic therapies and tumor progression, warranting further clinical development.

Jayson et al. [118] investigated HuMV833, a radiolabeled humanized monoclonal antibody targeting VEGF, for molecular imaging and biological evaluation. The antibody was labeled with ^124^I for PET imaging and ^125^I for biodistribution studies, achieving a radiochemical purity >95% and radiochemical yield of ~75%. In vitro assays confirmed that the radiolabeled HuMV833 maintained high VEGF binding (1.25 × 10^8^ M^−1^). The study aimed to assess HuMV833’s pharmacokinetics and tumor targeting to inform the design of clinical trials for anti-angiogenic therapies. The PET imaging study in a cancer patient revealed that tumor uptake varied between 16% and 75% during a 24 h period. PET imaging demonstrated high tumor-to-background contrast, with notable uptake in the liver (2.4 µg/mL) and kidneys (~2.8 µg/mL) due to antibody clearance. Blood clearance followed a biphasic pattern, with a biological half-life of 8–9 days, supporting the feasibility of HuMV833 for VEGF imaging and therapeutic monitoring; however, no clear relationship was observed between plasma pharmacokinetics and drug clearance from the tumor over 24–48 h. The study demonstrated that radiolabeled HuMV833 PET imaging could serve as a biomarker for patient stratification and therapy response assessment in anti-VEGF treatments, providing a rationale for its clinical development.

Christoforidis et al. [119] evaluated the intravitreal pharmacokinetics and biodistribution of ^124^I-labeled bevacizumab and ranibizumab using PET/CT imaging in a rabbit model. The radiolabeling process achieved a radiochemical purity >95%. Following intravitreal injection, PET imaging demonstrated a biphasic clearance pattern, with bevacizumab exhibiting a longer half-life (~4.2 days) compared to ranibizumab (~2.8 days), consistent with their molecular sizes and structures. The study confirmed that both antibodies remained localized in the vitreous humor for an extended duration, with minimal systemic distribution, supporting their long-acting effects in intraocular VEGF inhibition.

In this study, bevacizumab showed a slightly prolonged retention when compared with ranibizumab. The findings suggest that intravitreal PET imaging with ^124^I-labeled antibodies could provide valuable insights into drug distribution, clearance, and therapeutic efficacy for VEGF-targeting treatments in ocular diseases. This study supports the feasibility of PET/CT for tracking anti-VEGF agents in vivo, aiding in optimizing dosing regimens for clinical use.

Christoforidis et al. [120] conducted a detailed investigation into the systemic biodistribution and intravitreal pharmacokinetics of bevacizumab, ranibizumab, and aflibercept in a nonhuman primate model using ^124^I-radiolabeled tracers. The study achieved over 95% radiochemical purity for all radiolabeled compounds, ensuring the reliability of imaging and quantitative assessments. Intravitreal pharmacokinetic analysis revealed that bevacizumab had the longest vitreous half-life (~3.6 days), followed by aflibercept (~2.73 days) and ranibizumab (~2.44 days), correlating with molecular size and structural differences. PET/CT imaging demonstrated extended ocular retention of bevacizumab, whereas ranibizumab exhibited faster clearance, suggesting potential implications for treatment frequency and duration in clinical applications.

Systemic biodistribution studies showed minimal systemic exposure for all three agents, with <1.6 SUV detected in non-ocular organs, indicating limited off-target effects. Liver and kidney uptake were low but detectable, with aflibercept demonstrating slightly higher systemic retention when compared with bevacizumab and ranibizumab. These data provide critical insights into the pharmacokinetics of anti-VEGF therapies, aiding in optimizing dosing regimens while minimizing systemic exposure risks, particularly in patients requiring long-term treatment for retinal diseases.

#### 1.2.4. Nanoparticles and Novel Imaging Agents

Ebrahimi et al. [70] investigated a ^99m^Tc-radiolabeled anionic citric acid dendrimer conjugated with a VEGF antagonist peptide for breast cancer imaging and therapy monitoring. The team used a piece of software to optimize critical radiolabeling parameters and obtained the ^99m^Tc-labeled imaging agents at a high yield. The 3-(4,5-dimethylthiazol-2-yl)-2,5-diphenyltetrazolium bromide (MTT) assays demonstrated no toxicity in normal cells and dose-dependent toxicity in cancer cells. SPECT imaging of the ^99m^Tc-dendrimer-anti-VEGF in mice revealed high accumulation in the tumor region, supporting its specificity for tumor vasculature. There was also notable accumulation in the liver, suggesting the hepatobiliary system as the main excretion route. These results suggest that dendrimer-anti-VEGF could serve as an effective dual-functional agent for imaging and potential therapeutic application against breast cancer. The authors acknowledge that further studies are required to confirm its clinical potential, especially given its promising initial results in preclinical breast cancer models using the 4T1 cell line.

Shi et al. [121] presented a graphene oxide (GO)-based nanoconjugate labeled with ^64^Cu and functionalized with a VEGF121 (^64^Cu-GO-VEGF-121)-targeting ligand for precise tumor imaging. The radiolabeling process yielded high stability in serum, with over 95% of ^64^Cu retained on the GO-VEGF-121 nanoconjugates. In vivo PET imaging on U87MG tumor-bearing mice showed rapid and high tumor uptake, visible as early as 0.5 h p.i., peaking at 8.2 ± 1.4 %ID/g by 3 h. Biodistribution studies demonstrated a high tumor-to-muscle ratio (8.4 ± 2.1), more than twice that of non-targeted groups, indicating excellent targeting specificity over VEGF121. High liver uptake was observed initially (24.9 ± 3.0 %ID/g), decreasing over 48 h, suggesting predominant hepatobiliary clearance.

Additional assessments using histology and flow cytometry confirmed strong targeting specificity of ^64^Cu-GO-VEGF-121 to VEGFR-expressing tumor vasculature, with minimal non-specific binding. These findings underscore the potential use of ^64^Cu-GO-VEGF-121 as a highly stable, tumor-specific imaging agent capable of efficiently targeting VEGFR in the U87MG glioblastoma model, which is superior to the passive targeting strategy typically observed in current GO nanoconjugates.

Unlike PET or SPECT, microbubbles use non-ionizing radiation, which allows for safe repeated use and does not utilize radioactive materials. VEGFR-2-targeted microbubble (MBVEGFR-2) is used for ultrasound molecular imaging (USMI) to detect early cervical cancer in mice. Zhong et al. [122] reported that VEGFR-2-targeted USMI was able to detect cervical carcinoma less than 3 mm, proving its high sensitivity and suitability to distinguish solid tumors of angiogenesis and vascularization with no observed side effects in mice during dual intravenous injection. One limitation is that the sensitivity decreased as tumor size increased. The results showed its ability to detect lesions in the early stage and highlighted its imaging potential for future clinical applications. However, optimization of in vivo stability, biodistribution, and pharmacokinetics still needs to be studied before clinical translation.

**Figure 2:**
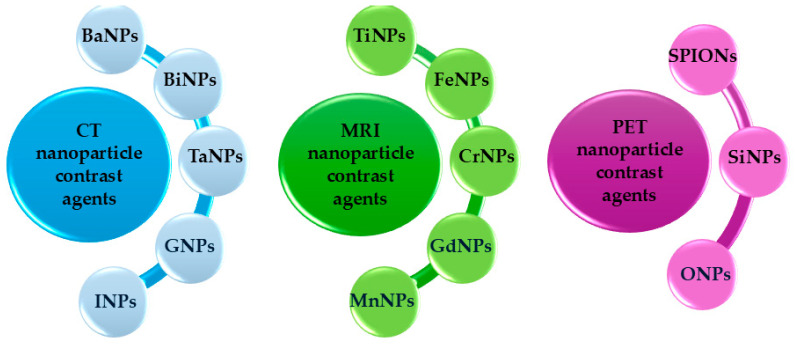
Schematic representation of various nano-based contrast agents utilized in medical imaging. Metal NPs, such as barium-based nanoparticles (BaNPs), bismuth-based nanoparticles (BiNPs), tantalum-based nanoparticles (TaNPs), gold-based nanoparticles (GNPs), and iodine-based nanoparticles (INPs), are depicted as contrast enhancers for computed tomography (CT) and titanium-based nanoparticles (TiNPs), iron-based nanoparticles (FeNPs), chromium-based nanoparticles (CrNPs), gadolinium-based nanoparticles (GdNPs), and manganese-based nanoparticles (MnNPs) are used as nano-based contrast agents for MRI imaging, providing high atomic number-based signal amplification. Radiolabeled nanoparticles, such as superparamagnetic iron oxide nanoparticles (SPIONs) and silica-based nanoparticles (SiNPs), as well as organic nanoparticles (ONPs), are illustrated for PET, enabling precise and quantitative visualization of molecular targets in vivo. This multimodal nanoparticle platform highlights the versatility of nanotechnology in noninvasive cancer diagnostics and monitoring [123,124,125].

While these novel nano-based imaging agents, Figure 2, offer significant benefits, each presents unique challenges that require further investigation to ensure long-term safety and establish standardized protocols. Nevertheless, integrating these advanced agents with traditional methods holds great promise to enhance biomarker detection, advancing VEGFR-dysfunction-associated disease diagnostics, treatment, and prognosis in clinical practice. A summary of radionuclide-based strategies for imaging and therapy of the VEGFR family is listed in Table 2.

## 2. Discussions and Perspective

Several FDA-approved drugs and imaging agents have been developed to target the VEGF-VEGFR system for both therapeutic and diagnostic purposes [130,131]. These agents have significantly improved outcomes in patients, although they are often associated with drug resistance mechanisms and off-target effects, necessitating precise patient selection and monitoring. The VEGFR family plays a critical role in lymphangiogenesis and vascular homeostasis, making it a key player in lymphatic disorders, including those affecting the brain lymphatic system [43]. VEGFR-3, expressed primarily in lymphatic endothelial cells, is crucial for the development and maintenance of the lymphatic vasculature [5,44]. Dysregulation of VEGF-C/VEGFR-3 signaling has been implicated in lymphedema and pathological lymphangiogenesis, which may contribute to neuroinflammatory and neurodegenerative diseases [132]. In the context of the brain, the glymphatic system, a recently identified perivascular network facilitating CSF drainage, relies on functional lymphatic vessels for waste clearance [133,134]. Impairments in VEGFR-mediated lymphatic function could lead to reduced CSF drainage, exacerbating conditions such as Alzheimer’s disease [135], multiple sclerosis [43], and glioma progression [136] due to the accumulation of toxic metabolites and immune dysregulation [7].

Recent research suggests that targeting the VEGFR family, particularly VEGFR-3, could provide novel therapeutic and imaging strategies for brain lymphatic dysfunction [64]. Modulating VEGF-C/VEGFR-3 signaling might enhance glymphatic clearance, potentially mitigating neuroinflammation and promoting brain homeostasis [137]. Additionally, VEGFR-2, which is traditionally associated with blood vessel angiogenesis, has been implicated in the crosstalk between vascular and lymphatic systems in the brain, influencing fluid balance and immune surveillance. Understanding the role of VEGFR-driven lymphangiogenesis in the central nervous system could pave the way for novel treatments for neurodegenerative diseases and brain tumors [138]. Further studies are needed to delineate the precise mechanisms by which VEGFR signaling impacts brain lymphatic function and to develop targeted therapies that restore proper lymphatic drainage in pathological conditions.

The use of radionuclides for imaging and therapeutic applications targeting VEGFR pathways has demonstrated significant potential in both preclinical and clinical settings. VEGFR-specific imaging agents, such as antibodies, peptides, and aptamers labeled with radionuclides like ^68^Ga, ^99m^Tc, and ^89^Zr, have provided precise visualization of tumor angiogenesis, lymphangiogenesis, and receptor expression patterns. Radiolabeled antibodies or peptides targeting VEGFR-3, a key receptor in lymphangiogenesis, enable noninvasive imaging of lymphatic vessel integrity and function using PET or SPECT. Additionally, fluorescent and contrast-enhanced MRI tracers targeting VEGF-C/VEGFR-3 interactions provide high-resolution imaging of lymphatic vessel dynamics, particularly in brain lymphatic networks [139]. These imaging strategies are crucial for detecting lymphatic dysfunction in neurodegenerative diseases, brain tumors, and inflammatory disorders, where impaired lymphatic drainage may exacerbate pathology [140]. Advances in molecular imaging probes specific to VEGF/VEGFR signaling hold promise for early diagnosis, treatment monitoring, and evaluating the therapeutic modulation of brain lymphatic function in various diseases [141].

The VEGF-VEGFR system is a keystone in the imaging and therapeutic landscape of various diseases due to its exceptional specificity, uniqueness, and binding potential. VEGFRs are primarily expressed on endothelial cells and are critically involved in angiogenesis, making them highly specific targets for diseases characterized by irregular vascular growth, such as cancer and age-related macular degeneration. The unique interaction between VEGF and VEGFR is governed by high-affinity binding, ensuring precise targeting and minimizing off-target effects [142]. This specificity not only enhances the accuracy of imaging modalities, such as PET and SPECT, but also improves the efficacy and safety profile of therapeutic interventions, including monoclonal antibodies and TKI. By using the special properties of the VEGF-VEGFR system, researchers and clinicians can achieve a dual benefit of early disease detection and targeted treatment, paving the way for personalized medicine approaches [6].

Despite these advancements, several limitations remain. The heterogeneity of VEGFR expression, challenges in tumor penetration, and variability in radionuclide pharmacokinetics can impact the efficacy of imaging and therapy. Off-target accumulation, potential toxicity, and the need for sophisticated radiochemistry infrastructure further complicate the clinical translation of some radiopharmaceuticals. Additionally, the relatively high cost and regulatory hurdles of radionuclide-based agents are significant barriers to widespread adoption. Large molecules, such as antibodies, have slow clearance that can limit image contrast, while smaller molecules have suboptimal tumor retention [96].

To overcome current limitations relating to VEGFR imaging, future research needs to improve the specificity and pharmacokinetics of targeting strategies. These improvements include combining computational docking to increase hit success rate, establishing reliable kinetic modeling and imaging analysis, incorporating rigorous animal models, and using test–retest to increase reproducibility [143]. To provide a more comprehensive view of tumor angiogenesis, the exploration of multimodal imaging techniques and validation of imaging biomarkers is needed to optimize the use of VEGF imaging for clinical use [15]. Moreover, the incorporation of higher species in nonhuman primates, a large sample size in clinical translation, and overcoming polymorphism issues are needed to test the validity of the imaging in various cancer types and treatment settings.

PET and SPECT imaging techniques provide a non-invasive method to assess VEGFR expression and monitor the response to anti-angiogenic therapies [144]. Nuclear medical imaging provides a way to stratify patients and optimize treatment for various cancer types. When PET and SPECT for VEGFR-targeted studies are evaluated, both modalities offer distinct advantages and limitations, with their suitability largely depending on the research or clinical context. PET excels in sensitivity, spatial resolution, and quantitative accuracy, making it particularly well-suited for detailed imaging of VEGFR expressions and dynamic processes such as angiogenesis and lymphangiogenesis. Commonly used PET radionuclides, including ^18^F, ^68^Ga, ^64^Cu, and ^89^Zr, enable high-resolution, real-time visualization with shorter imaging times. In contrast, SPECT, employing radionuclides like ^99m^Tc and ^111^In, is more accessible and cost-effective, making it a practical choice in resource-limited settings. However, its lower resolution and sensitivity may hinder the detection of subtle changes in VEGFR expression [130]. Overall, PET is often preferred for VEGFR studies requiring precision and quantification, while SPECT remains a practical alternative for broader diagnostic applications. While VEGFR-targeted imaging agents are less common than therapeutic drugs, several agents and strategies have been explored for monitoring angiogenesis and VEGFR expression in clinical investigations. [^89^Zr]bevacizumab is an FDA-approved antibody used for immuno-PET by assessing VEGF expression levels in humans [145]. Currently, there are over 100 active clinical trials using VEGF-targeting agents [146]. However, predominant clinical trials are focused on therapeutic evaluation and most nuclear medicine imaging agents moved to clinical investigation are still bevacizumab-based [69,110,111,147]. Therapeutic radionuclides, including ^177^Lu, ^225^Ac, and ^90^Y, offer targeted radiotherapy options that deliver localized cytotoxic effects, minimizing damage to surrounding healthy tissues [72], but clinical investigations involving these therapeutic radionuclides targeting the VEGF-VEGFR family are still emerging. Looking ahead, advancements in hybrid systems such as PET/SPECT or PET/MRI and the development of well-designed specific tracers are poised to further enhance the capabilities and comparative utility of these imaging modalities in VEGFR research [148].

## 3. Conclusions

While challenges remain, the progress in VEGFR-targeted radionuclide imaging and therapy underscores their importance as tools for cancer diagnosis, monitoring, and treatment. Continued innovation and collaboration between researchers, clinicians, and industry stakeholders will be pivotal in overcoming current limitations and unlocking the full potential of radionuclide-based approaches in VEGF-associated disorders.

## Figures and Tables

**Table 1:** Overview of the VEGF-VEGFR system and its roles in disease conditions.

VEGFR	Ligand (s)	Disease Conditions	Role in Disease	Broad or Specific Role *	Ref.
VEGFR-1	VEGF-A, VEGF-B, PlGF	- Cancer: Tumor growth and metastasis - Cardiovascular diseases: Atherosclerosis, ischemia	Acts as a decoy receptor modulating VEGF-A availability; drives angiogenesis and inflammatory responses.	Broad	[49,50]
VEGFR-2	VEGF-A, VEGF-C, VEGF-D	- Cancer: Solid tumors - Diabetic retinopathy	Central driver of angiogenesis, vascular permeability, and endothelial cell proliferation.	Broad	[8,51,52]
VEGFR-3	VEGF-C, VEGF-D	- Lymphedema: Primary lymphedema, Milroy disease - Neurological diseases: Alzheimer’s disease, multiple sclerosis, traumatic brain injury, epilepsy	Regulates lymphangiogenesis and meningeal lymphatic function, influencing waste clearance, immune cell trafficking, and fluid drainage.	Specific to lymphatic and neuroimmune roles	[5,53,54]
VEGFR-1 and VEGFR-2	VEGF-A	- Cancer: Tumor progression and metastasis - Ischemic diseases: Myocardial infarction, stroke	Orchestrated roles in pathological angiogenesis; VEGFR-1 modulates VEGFR-2 signaling to balance vascular growth.	Broad	[55,56,57]
VEGFR-3	VEGF-C, VEGF-D	- Cancer: Lymphatic metastasis - Lymphatic disorders: Secondary lymphedema	Promote tumor lymphangiogenesis, immune modulation, and waste clearance; critical in metastasis and fluid homeostasis.	Broad	[58,59,60]
VEGFR-1	PlGF	Preeclampsia - Cardiovascular diseases: Heart failure, hypertension	PlGF enhances VEGFR-1 signaling, exacerbating inflammation and vascular dysfunction in conditions like preeclampsia and ischemia.	Specific to vascular inflammation	[61]
VEGFR-2	VEGF-A	Ocular diseases: Wet age-related macular degeneration (AMD), diabetic retinopathy	Overactivation induces pathological angiogenesis and vascular leakage, driving vision loss in retinal diseases.	Specific to ocular diseases	[62]
VEGFR-3	VEGF-C, VEGF-D	- Cancer metastasis: Through lymphatic spread	Regulates lymphatic vessel growth and function; overexpression supports cancer cell dissemination and immune evasion.	Specific to lymphatic roles	[63,64]

* This column outlines the functions of VEGF receptors (VEGFR-1, VEGFR-2, and VEGFR-3), their primary ligands, associated diseases, and their contributions to pathophysiology. Some receptors are involved in multiple biological systems and contribute to a wide range of conditions. For example, VEGFR-1 and VEGFR-2 regulate angiogenesis, vascular permeability, and inflammation across various cancers, cardiovascular diseases, and ocular disorders. In contrast, others have more restricted functions in specific tissues or pathological contexts. VEGFR-3, for instance, plays a critical role in the development and function of the lymphatic system and is primarily implicated in lymphedema, lymphatic metastasis, and neurological disorders where lymphatic clearance is essential. This differentiation is crucial for developing precision therapies aimed at either widespread vascular regulation or highly localized disease mechanisms, such as retinal angiogenesis or lymphatic dysfunction.

**Table 2:** Radionuclide-based strategies for imaging and treatment of VEGFR family-associated diseases.

Imaging /Therapy/Stage *	Compound	Targeting Agent	Application	Advantages	Disadvantages	Ref.
SPECT/CT/Preclinical	Antibody	[^99m^Tc]Tc MAG3-bevacizumab	Atherosclerotic cardiovascular	1. Specific imaging of neovascularization in atherosclerotic plaques 2. Correlation with histopathology	1. Repeated administration for imaging 2. Plaque heterogeneity not fully addressed	[102]
SPECT/Preclinical	Antibody	[^99m^Tc]Tc-HYNIC-chtiosan-Cy5.5-DC101	Ischemic microvasculature	High specificity, improved stability, potential for dual functionality	Complexity in synthesis, possible degradation in vivo	[103]
SPECT/Preclinical	Antibody	[^99m^Tc]Tc-HYNIC-BV, [^99m^Tc]Tc(CO)_3_-BV,[^99m^Tc]Tc-DTPA-BV	Melanoma	Versatile radiolabeling, in vitro and in vivo stability, reduced immunogenicity	Complex synthesis and purification	[104,105,106]
SPECT/Preclinical	Antibody	[^99m^Tc]Tc-Ram	VEGFR-2 receptor	High target specificity, theranostic potential, enhanced stability	Complex radiolabeling, non-specific uptake	[107]
SPECT/CT/Preclinical	Nanoparticle	[^99m^Tc]Tc-dendrimer-anti-VEGF	Breast cancer	1. High specificity for VEGF, which could enhance tumor targeting. 2.The ^99m^Tc labeling makes it suitable for clinical imaging 3. Dendrimer-based probes can offer highly efficient drug delivery and reduced toxicity	1. The specificity of the probe to VEGF in other tissues and tumors needs further investigation 2. Limited data on the long-term stability, circulation time, and biodistribution. 3. Challenges in large-scale clinical production	[70]
PET/CT/Preclinical, Clinical	Antibody	[^111^In]In-DTPA-BV	Colorectal cancer, ovarian tumor	Established radiochemistry, potential for monitoring therapy, safety of bevacizumab, clinical relevance	Poor correlation with VEGF-A expression, non-specific uptake	[69]
SPECT/CT/Preclinical	Affibody	[^111^In]In-NODAGA-Z_VEGFR-2_-Bp2	GBM	High specificity, improved tumor penetration, rapid clearance, customizable affibody design, reduced immunogenicity	Limited tumor specificity for VEGFR-2	[95]
PET/Preclinical	Small molecule	[^18^F]su11248	Tyrosine kinase activity in cancer	High specificity, high resolution and sensitivity, favorable isotope properties, potential for early diagnosis	Complex synthesis, potential off-target effects, short half-life of ^18^F, competition with endogenous ligands	[78]
PET/Preclinical	Small molecule	[^18^F]3-[4′-Fluorobenzylidene]indolin-2-one	RTKs	Potential for broad cancer applications, facilitates personalized medicine	Complex synthesis, limited tumor specificity, off-target toxicity	[79]
PET/Preclinical	Small molecules	[^18^F]F-diaryl urea	Angiogenesis	Development of dual inhibitors, specificity for angiogenesis-related targets, potential for personalized medicine	Potential off-target effects, cost and technical barriers	[80]
PET/CT/Preclinical	Antibody	[^64^Cu]Cu-NOTA-BV	Renal carcinoma	Innovative use of immuno-PET, rapalog therapeutic monitoring, enhanced specificity	Potential for off-target effects	[12]
PET and NIRF/Preclinical	Antibody	[^64^Cu]Cu-NOTA-BV-800CW	GBM	Dual-modality imaging, real-time surgical guidance	High cost and limited accessibility, technical complexity	[108]
PET/Preclinical	Antibody	[^64^Cu]Cu-NOTA-RamAb	Lung cancer	Targeted imaging of VEGFR-2, high sensitivity and quantification, potential for therapy monitoring, clinical translation	Off-target accumulation	[109]
PET/Preclinical	Peptide	[^64^Cu]Cu-DOTA-GU40C4	Prostate cancer	High stability, versatility of peptoid structure binding to VEGFR-2 and simplicity	Binding affinity concerns	[87]
PET/Preclinical	Nanographene	[^64^Cu]Cu-NOTA-GO-PEG-VEGF-121	Brain Cancer	1. GO’s large surface area enabled functionalization with targeting agents and therapeutic payloads, enhancing its versatility 2. Reducing off-target effects 3. Conducted in vivo studies using a xenograft model to confirm tumor accumulation 4. GO serves as a platform for both tumor imaging and therapeutic delivery, offering multimodal capabilities 5. VEGFR targeting improves tumor vascular interaction, enhancing therapeutic delivery	1. Despite increased tumor specificity, non-specific high accumulation in the liver and spleen was noted, a common issue with nanoparticles 2. No evaluation of BBB permeability 3. The long-term stability of the functionalized GO in biological systems was not reported	[121]
PET/Preclinical	Peptide	[^64^Cu]Cu-DOTA-VEGF_125-136_	Melanoma	1. Demonstrated excellent binding affinity for VEGFR as shown by significant signal reduction in blocking studies (>90%), confirming target-mediated uptake 2. Rapid tumor accumulation and imaging 3. exhibited rapid clearance from blood (20 min) and low non-target tissue uptake, minimizing background signals and improving tumor-to-background contrast	1. Rapid clearance reduced imaging windows and may necessitate precise timing. 2. Single receptor targeting may limit its utility in tumors with heterogeneous or low VEGFR expression	[10]
PET/Clinical	Antibody	[^89^Zr]Zr-N-suc-Df-BV	Breast cancer, lung cancer	Early detection, therapeutic monitoring, potential for combination therapies	Limited sample size, complexity of interpretation, high cost and limited accessibility	[110,111]
PET and NIRF/Preclinical	Antibody	[^89^Zr]Zr-N-suc-Df-BV/cetuximab-800CW, [^89^Zr]Zr-N-suc-Df-BV/cetuximab	Squamous cell carcinoma	Dual-mode imaging, inert coupling method, versatility for preclinical and clinical applications, improved sensitivity, enhanced targeting	Potential for high background signal in fluorescence	[112,113]
PET/CT/Preclinical	Antibody	[^89^Zr]Zr-N-suc-Df-Ram	Prostate cancer	Specific targeting of VEGFR-2, immuno-PET technology, clinical applicability, alignment of biological half life of intact Ab to ^89^Zr half life	Slow blood clearance, longer imaging window	[114]
PET/CT/Preclinical	Antibody	[^89^Zr]Zr-bevacizumab, [^89^Zr]-IgG	Ovarian Tumor	1. High affinity for VEGF, ensuring precise imaging of VEGF-overexpressing tumors 2. Bevacizumab is an FDA-approved drug, facilitating potential clinical adaptation	1. Stability of compound in vivo was not comprehensively assessed 2. Extended circulation time may increase non-specific background signal	[69]
PET/CT/Clinical	Antibody	[^89^Zr]Zr-bevacizumab	Renal Cell Carcinoma	1.The use of a clinically available radiotracer (^89^Zr-bevacizumab) allows for translation into clinical practice 2. Early detection of therapy efficacy could lead to more personalized treatment plans, improving patient outcomes	1. The study is limited by its small patient cohort 2. PET imaging using ^89^Zr-bevacizumab might not be suitable for all tumor types, limiting its applicability 3. Specificity and sensitivity in a heterogeneous patient population might vary, requiring further validation	[92]
SPECT/MRI/Clinical	Antibody	[^123^I]I-VEGF165	Braintumor	1. Non-invasively assess VEGF expression and angiogenesis in glioma patients 2. A significant correlation between the tumor-to-normal brain (T/N) uptake ratio and overall survival 3. Specificity for high-grade gliomas	1. A total of 23 patients, with only 8 undergoing both imaging modalities. 2. Limited temporal imaging	[116]
SPECT/Clinical	Antibody	[^123^I]I-VEGF	Glioblastoma (GBM)	1. ^123^I-VEGF provides an effective method for visualizing brain tumors, particularly those with significant angiogenesis. 2. Scintigraphy using ^123^I offers compatibility	1. More studies are required to investigate the probe’s specificity for tumors and non-specific uptake in other organs/tissues 2. More information is needed on dosimetry, circulation time, and stability in clinical contexts, particularly for brain tumors where BBB disruption may play a role	[126]
PET/Preclinical	Antibody	[^124^I]I-VG76e	Fibrosarcoma	High specificity, potential for therapy monitoring, use of iodine-124, potential for therapy monitoring	Challenges with iodine-124 labeling, tracer stability and deiodination, biodistribution challenges	[117]
Therapy/PET/CT/MRI/Clinical	Antibody	[^124^I]I-HuMV833	Tumorendothelial permeability	Comprehensive evaluation of HuMV833, promise for therapy monitoring, guidance for antiangiogenic trials, dual approaches of imaging and biology	Immunogenicity concerns, inadequate addressing of resistance mechanisms	[118]
PET/CT/Preclinical	Antibody	[^124^I]I-Ran, [^124^I]I-BV	Pharmacokinetic properties ofvitreous cavity	Innovative use of radiolabeled antibodies, relevant to ocular diseases, quantitative assessment, potential for clinical translation	High cost and complexity of radiolabeling, short follow-up period, lack of functional assessment	[119]
PET/CT/Preclinical	Peptide	[^124^I]I-aflibercept	Vitreous cavity	Comprehensive comparison of three anti-VEGF agents, use of a nonhuman primate model, relevant to ocular therapeutics, addresses regulatory and therapeutic concerns	Cost considerations, limited long-term data, variability in drug dosing, limited exploration of resistance mechanisms	[120]
Ultrasound/Preclinical	Antibody	[^125^I]MBs-I-Bt-Avas12a1	Angiosarcoma tumor	Novel use of targeted microbubbles, high sensitivity and specificity for VEGFR-2, non-ionizing modality, potential for real-time imaging, economic and accessibility benefits	Limited depth of ultrasound, microbubble stability issues	[16]
SPECT/Preclinical	Small molecule	[^125^I]5-I-sunitinib	Angiogenetic process	Integration of radiochemistry and pharmacology, efficient radiosynthesis protocol, exploration of VEGFR targeting, broad applicability	Limited clinical relevance of I-125, complexity of radiochemistry, no comparison with existing radiotracers	[81]
SPECT/CT/Preclinical	Small molecule	[^125^I]m-I-NPAE, [^125^I]p-I-NPAE[^125^I]m-I-NPAM[^125^I]p-I-NPAM	Prostate cancer	Insights into tumor angiogenesis, potential for multi-disease applications	Potential toxicity of derivatives, short-term evaluation	[82]
PET/Preclinical	Small molecule	[Methyl-^11^C]-sorafenib	Head and neck cancer	Focus on brain uptake, innovative use of knockout models, efficient radiosynthesis, potential clinical translation, contribution to drug transport studies	No functional imaging data, absence of comparison, radiochemical yield and stability concerns	[77]
PET/Preclinical	Small molecule	[N-Methyl-^11^C]-PAQ	Subcutaneous and intraperitoneal tumor models	Potential for cancer therapy monitoring, targeted imaging for VEGFR-2	Potential for non-specific binding	[83]
Synthesis/Preclinical	Small molecule	[N-Methyl-^11^C]vandetanib, [N-methyl-^11^C]chloro-vandetanib,[O-methyl-^11^C]vandetanib[O-methyl-^11^C]chloro-vandetanib	Tumor angiogenesis	Efficient radiosynthesis protocols, dual tracer development, potential for multitarget imaging, broad applicability	No functional imaging data, lack of tumor models, no pharmacokinetic data	[84]
PET/Preclinical	Antibody	[^68^Ga]Ga-NOTA-VEGF-121	Brain Cancer	1. High affinity and specificity of NOTA-VEGF-121 for VEGFR-2 (IC_50_ = 1.66 nM), ensuring selective imaging of VEGFR-rich tumors 2. Readily accessible to ^68^Ga 3. High tumor-to-background contrast, facilitating accurate localization of VEGFR expression. 4. Rapid renal clearance minimized non-specific background signal, enhancing imaging quality	1. The in vivo stability of radiolabeled compound was not extensively reported 2. No evaluation of probe uptake in CNS tumors with an intact BBB, limiting its application to glioblastomas with disrupted barriers 3. The rapid clearance of the probe may limit imaging windows and necessitate precise timing for imaging sessions	[86]
PET/CT/Preclinical/Clinical	Peptide	[^68^Ga]Ga –DOTA-TMVP1	Ovarian, Cervical cancer	Improved tumor targeting for gynecological cancers due to high VEGFR-3 expressions	1. Probe uptake in non-tumor tissues were not fully evaluated 2. The circulation time and dosimetry need further investigation	[127]
PET/CT/Preclinical	Pepetide	[^68^Ga]Ga –DOTA-TMVP1448	Tumor metastatic lymph node	1. TMVP1448 shows high specificity for VEGFR-3 (K_D_ = 6.73 × 10^−6^ mol/L). 2. The peptide inhibitor allows for better tumor penetration and retention	More pre-clinical study, including biodistribution and dosimetry, is required to support its potential for clinical investigation	[128]
Therapy/Preclinical	Peptide	[^177^Lu]Lu -DOTA-Ahx-A7R,[^177^Lu]Lu Lys(hArg)-Dab(Ahx-DOTA-Lu)-Pro-Arg,[^68^ Ga]Ga -DOTA-Ahx-A7R,[^68^ Ga]Ga Lys(hArg)-Dab(Ahx-DOTA-Lu)-Pro-Arg	Cancer therapy	1. Dual-isotope theranostic design. 2. The branched peptidomimetic exhibited stronger binding to NRP-1 than the linear A7R peptide	1. Poor stability in human serum 2. Lack of in vivo evaluation	[88]
Therapy/Preclinical	Antibody	[^177^Lu]Lu-CHX-A”-DTPA-BV	Lymphoma and acute myeloid leukemia	Efficient radiolabeling with CHX-A’’-DTPA, demonstrated therapeutic potential	Pharmacokinetics of bevacizumab with limited extravascular distribution, immunogenicity concerns	[115]
Therapy/Preclinical	Antibody	[^177^Lu]Lu-DTPA-Anti- VEGFR-1	Lung carcinoma	1. Dual benefits of targeted radiotherapy and imaging due to β-particle emission and γ-photons 2. Minimizing off-target effects 3. Effective radiolabeling and binding specificity, supporting further in vivo evaluations	1. In vivo xenograft or transgenic tumor models are not included 2. No analysis of BBB permeability 3. Non-specific uptake in non-target tissues was not thoroughly evaluated. 4. No exploration of the pharmacokinetics or circulation half-life	[129]
Therapy/Preclinical	Antibody	[^177^Lu]Lu-cG250	Renal Cell Carcinoma	1. Complementary mechanisms of action—anti-angiogenic effects of sunitinib and targeted cytotoxicity of ^177^Lu-cG250 radioimmunotherapy (RIT)—to improve therapeutic outcomes in RCC, particularly in resistant tumors 2. Proved effective in the sunitinib-resistant SK-RC-52 model, showing significant tumor growth delay and survival benefit (91% survival with two cycles) 3. Histopathological confirmation	1. Lack of functional imaging 2. While survival and tumor response were analyzed, the study offers limited detailed evaluation of long-term radiotoxicity or potential damage to normal organs beyond basic survival and histology	[94]

* Preclinical refers to studies that do not involve human subjects, while clinical refers to studies that involve human subjects.

## Data Availability

The materials and resources in this study are available from the corresponding author upon request.

## Abbreviations

The following abbreviations are used in this manuscript:

VEGFR	Vascular endothelial growth factor receptors
VEGF	Vascular endothelial growth factor
PlGF	Placental growth factor
PET	Positron emission tomography
SPECT	Single-photon emission computed tomography
MAPK	Mitogen-activated protein kinase
CSF	Cerebrospinal fluid
MRI	Magnetic resonance imaging
BBB	Blood–brain barrier
MW	Molecular weight
TKI	Tyrosine kinase inhibitors
HAECs	Human aortic endothelial cells
ID	Injected dose
PCR	Polymerase chain reaction
GMP	Good manufacturing practice
BFCA	Bifunctional chelating agent
DTPA-NCS	Isothiocyanatobenzyl-DTPA
DOTA	1,4,7,10-tetraazacyclododecane-1,4,7,10-tetraacetic acid
NOTA	1,4,7-triazacyclononane-1,4,7-triacetic acid
PEG	Polyethylene glycol
PBS	Phosphate-buffered saline
ELISA	Enzyme-linked immunosorbent assay
RIT	Radioimmunotherapy
WHO	World Health Organization
OS	Overall survival
IgG	Immunoglobulin
HUVECs	Human umbilical vein endothelial cells
NODAGA	1,4,7-triazacyclononane-1-glutamic acid-4,7-diacetic acid
ITLC	Instant thin-layer chromatography
HPLC	High-performance liquid chromatography
NIRE	Near-infrared fluorescence
NSCLC	Non-small cell lung cancer
MTT	3-(4,5-dimethylthiazol-2-yl)-2,5-diphenyltetrazolium bromide
USMI	Ultrasound molecular imaging
RCC	Renal cell carcinoma
MTT	3-(4,5-dimethylthiazol-2-yl)-2,5-diphenyltetrazolium bromide
HCC	Hepatocellular carcinoma
RGD	Arginyl-glycyl-aspartic acid
AMD	Age-related macular degeneration
Da	Daltons
IC_50_	Half-maximal inhibitory concentration
%ID/g	Percent injected dose per gram of tissue
MVD	Micro vessel density
CRC	Prostate cancer
SUVmax	Maximum standardized uptake value
CRC	Colorectal cancer
NIRF	Near-infrared fluorescence
